# Discovery of (meth)acrylate polymers that resist colonization by fungi associated with pathogenesis and biodeterioration

**DOI:** 10.1126/sciadv.aba6574

**Published:** 2020-06-05

**Authors:** Cindy Vallieres, Andrew L. Hook, Yinfeng He, Valentina Cuzzucoli Crucitti, Grazziela Figueredo, Catheryn R. Davies, Laurence Burroughs, David A. Winkler, Ricky D. Wildman, Derek J. Irvine, Morgan R. Alexander, Simon V. Avery

**Affiliations:** 1 School of Life Sciences, University of Nottingham, Nottingham, UK.; 2 School of Pharmacy, University of Nottingham, Nottingham, UK.; 3 Faculty of Engineering, University of Nottingham, Nottingham, UK.; 4 Advanced Data Analysis Centre, University of Nottingham, Nottingham, UK.; 5 Monash Institute of Pharmaceutical Sciences, Monash University, Australia.; 6 La Trobe Institute for Molecular Science, La Trobe University, Australia.; 7 CSIRO Manufacturing, Clayton, Australia.

## Abstract

Fungi have major, negative socioeconomic impacts, but control with bioactive agents is increasingly restricted, while resistance is growing. Here, we describe an alternative fungal control strategy via materials operating passively (i.e., no killing effect). We screened hundreds of (meth)acrylate polymers in high throughput, identifying several that reduce attachment of the human pathogen *Candida albicans*, the crop pathogen *Botrytis cinerea*, and other fungi. Specific polymer functional groups were associated with weak attachment. Low fungal colonization materials were not toxic, supporting their passive, anti-attachment utility. We developed a candidate monomer formulation for inkjet-based 3D printing. Printed voice prosthesis components showed up to 100% reduction in *C. albicans* biofilm versus commercial materials. Furthermore, spray-coated leaf surfaces resisted fungal infection, with no plant toxicity. This is the first high-throughput study of polymer chemistries resisting fungal attachment. These materials are ready for incorporation in products to counteract fungal deterioration of goods, food security, and health.

## Teaser

Anti-attachment materials that are sprayable and 3D-printable passively prevent colonization by harmful fungi.

## INTRODUCTION

The capacity for fungi to cause disease, spoilage, and biodeterioration is a major scourge for society. Fungal infections of humans are associated with high mortality rates (~50% in hospitalized patients), killing more than 1.5 million people annually (*1*, *2*). Fungi also destroy crops and postharvest foods sufficient to feed 600 million people annually (*3*, *4*). This has spawned the development of major antifungal and fungicide industries with a combined worth of ~$30 billion globally, even without accounting for fungicides used to tackle fungal biodeterioration of valuable products and materials. Antifungal drugs and fungicides provide our first line of defense against fungi. However, efficacy of the current arsenal of approved agents is being eroded by drug resistance (*2*). The issues of resistance, tightening antifungal/fungicide regulations, and mounting concerns for human and environmental health issues resulting from excessive chemical use have combined to underscore the need for alternative, sustainable strategies for fungal control.

Fungi need to attach to surfaces, both biological (e.g., epithelia and leaf surfaces) and inert (e.g., medical devices and household surfaces), to initiate many of the problems that they cause. Furthermore, in some scenarios (e.g., human infection) attached fungi can form biofilms, communities bounded by a biomaterial matrix. This property plays a crucial role in fungal virulence (*5*, *6*). Therefore, limiting attachment of fungal cells or spores to surfaces is a key but relatively unexploited target for combatting fungal colonization. Most strategies for tackling fungi rely on antifungals and fungicides, often incorporated into or applied to surfaces. In the case of surface colonization by human pathogens, “lock” therapy is used to eradicate biofilm formation on catheters before their contact with patients. This involves pretreating the devices with high concentrations of antifungal drug (*7*, *8*). Medical devices can also be coated or impregnated with inhibitory agents (*9*). To control fungal phytopathogens in agriculture, fungicides are commonly sprayed onto crops, but resistance is a major concern here. Another chemical-based crop protection strategy is the use of actives that perturb attachment, cell-to-cell communication, or dispersion without necessarily killing the fungi. The plant-derived bioactive zosteric acid, which alters oxidative balance of cells by targeting the NADH:quinone reductase (*10*, *11*), has been shown at sublethal concentrations to reduce adhesion of the phytopathogens *Magnaporthe grisea* and *Colletotrichum lindemuthianum* (*12*) and food spoilage fungi *Aspergillus niger* and *Penicillium citrinum* (*13*). These strategies all rely on the use of bioactive agents that, as outlined earlier, hold diminishing appeal for long-term fungal control. However, since fungal attachment is essentially a passive process, it is reasoned that passive approaches could provide effective control of fungi at the crucial surface attachment step. A passive intervention like an attachment-resistant material should exert less selective pressure for resistance than bioactive drugs, for example. This is because nonresistant fungi would not be killed by an anti-attachment surface, and development of resistance would typically require a gain of new function (i.e., ability to attach). Despite these advantages, such materials are difficult to design rationally because of our limited mechanistic knowledge of how fungi interact with different surfaces.

Several of the above issues also apply to the control of bacteria, which are evolutionarily distant from the fungi. An active-free approach has been developed recently, which shows promise for control of pathogenic bacteria (e.g., *Pseudomonas aeruginosa*). Specifically, using polymer microarrays (*14*), a copolymer of ethylene glycol dicyclopentenyl ether acrylate (EGDPEA) and di(ethylene glycol) methyl ether methacrylate (DEGMA), i.e., poly(EGDPEA-*co*-DEGMA), was developed following a high-throughput screen for bacterial anti-attachment properties among a large panel of monomers (*15*). The copolymer decreased bacterial biofilm formation when coated to silicone urinary catheters, compared with existing commercial catheters coated with silicone rubber and silver-containing hydrogel (*15*–*17*). Clinical trials in Europe are currently underway using catheters coated with this anti-attachment polymer. This resistance of the developed material to bacterial attachment has been attributed to their combined weakly amphiphilic nature and the molecular rigidity of the polymers’ pendant groups (*18*, *19*). The use of the high-throughput screening approach allowed this discovery of the novel polymer without requiring in-depth understanding of the underlying biological-material interactions. More recently, data mining of the structural characteristics exhibited by these resistant polymers has led to the identification of a predictive tool called the alpha parameter that has allowed the first “designed” resistant polymer to be synthesized from first principles (*19*, *20*).

A similar anti-attachment approach has not previously been reported for fungi despite the fact that key human pathogens such as *Candida albicans* avidly form biofilms, including on biomedical devices. Fungal biofilms necessitate replacement of expensive indwelling devices like voice prostheses every few months (*21*). Moreover, a passive anti-attachment technology for fungi could have far wider applications such as food security and longevity of commercial materials, which are substantially affected by fungal attack. It is not obvious that anti-attachment materials developed against bacteria, such as poly(EGDPEA-*co*-DEGMA), should also be effective against fungi. Bacteria and fungi have very different cell surface characteristics, e.g., their cell walls comprise distinct major components, in peptidoglycan and chitin, respectively.

In this study, we apply an acrylate/methacrylate-based polymer microarray screening approach (*15*) to fungi, to identify fungal anti-attachment materials. We characterize a group of polymeric materials that substantial reduce the attachment and biofilm formation of key fungi onto diverse surfaces [e.g., plant surfaces and three-dimensional (3D)–printed parts of medical devices], subject of a U.K. patent application (GB2002011.1). We demonstrate the potential of these materials to combat pasand sively fungal colonization and the possibility to 3D print medical devices from anti-attachment materials.

## RESULTS

### Identification of candidate fungal anti-attachment polymers by microarray screening

**Fig. 1:**
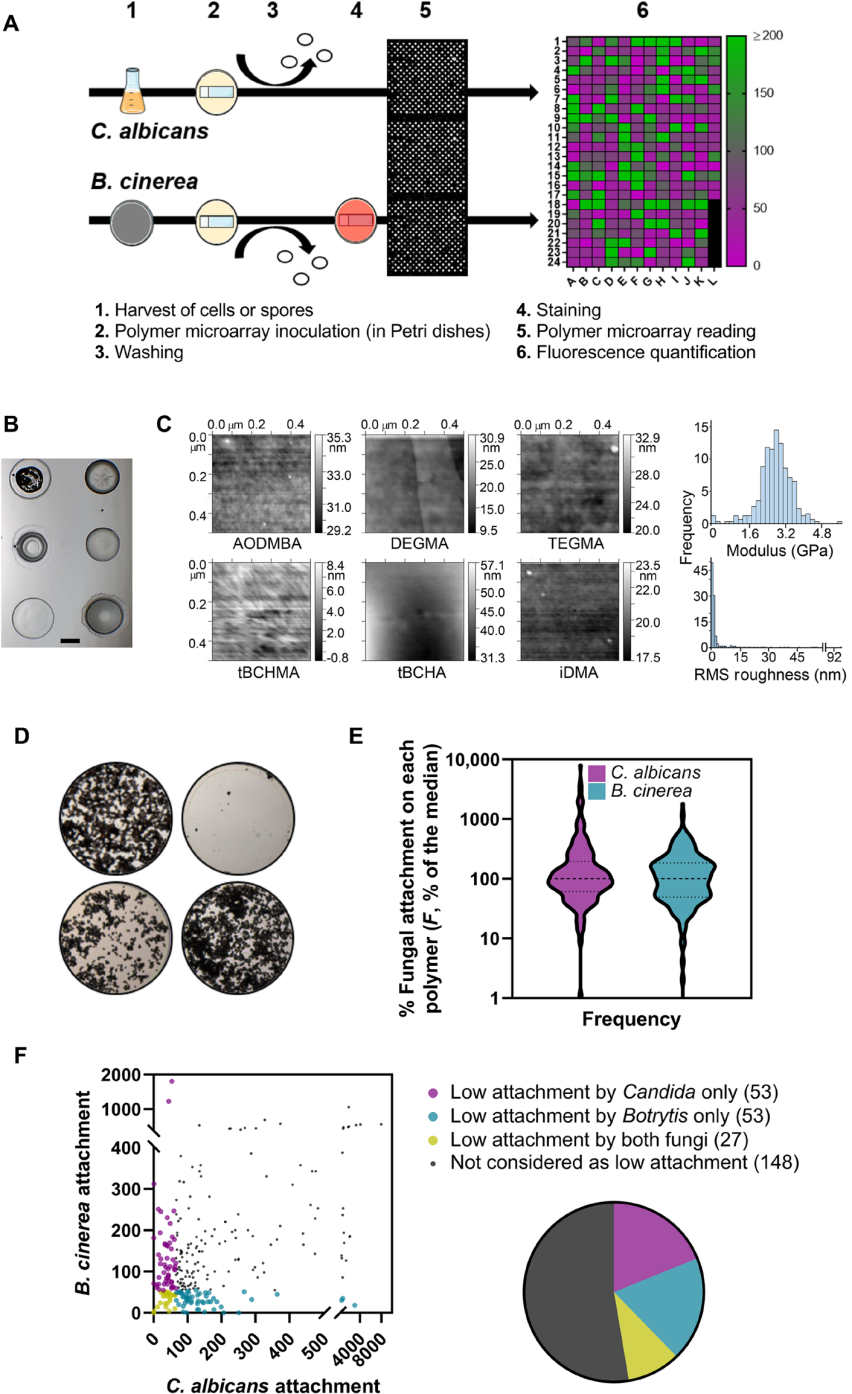
Polymer microarray screening for fungal attachment. (**A**) Fungal attachment assay procedure for *C. albicans* and *B. cinerea*, detected by yCherry expression or Congo red staining, respectively. (**B**) Microscopic image of a representative area encompassing six polymer spots on a polymer microarray slide. Scale bar, 150 μm. (**C**) Representative AFM height images of polymer microarray spots for six selected polymers (lead materials for *C. albicans*; see later) and histograms of modulus (dry) and roughness across all array polymers. AODMBA, (R)-α-acryloyloxy-β,β-dimethyl-γ-butyrolactone; TEGMA, tri(ethylene glycol) methyl ether methacrylate; tBCHMA, *tert*-butylcyclohexyl methacrylate; (**D**) Microscopic images of *B. cinerea* spore adhesion on glass (top left) and three representative microarray polymers with differing attachment properties. Top right: Polymer of interest. Polymer spots are ~300 μm in diameter. (**E**) Distribution of fungal attachment results across the polymer arrays. Attachment percentages are relative to the median (equal to 100%) for each fungus. Values are means from six (*C. albicans*) or three (*B. cinerea*) independent replicates. The full data and polymer names are listed in table S1. (**F**) Comparison of % attachment (relative to median) by *B. cinerea* and *C. albicans* to the different polymers. *R*^2^ = 0.097; *P* < 0.0001.

To identify materials that may resist fungal attachment, we screened 281 acrylate and methacrylate homopolymers printed in a microarray format (Fig. 1A). These encompassed bacterial anti-attachment candidates described previously (*15*) and other commercially available monomers that exhibited a wide chemical diversity within the side chains attached to the polymer backbone (table S1). Polymer spots were evenly distributed on arrays (Fig. 1B). Atomic force microscopy (AFM) images for selected materials are presented in Fig. 1C. Analysis by AFM of all array materials (Fig. 1C, right) showed that 90% had a root mean square (RMS) roughness less than 4.0 (including the six presented examples) and a mean modulus of 2.8 ± 0.7 GPa. Fungal attachment was determined after incubating suspensions of cells (*C. albicans*, yCherry-tagged) or spores (*Botrytis cinerea*, Congo red–stained) with the polymer microarrays for 2 or 6 hours, respectively (Fig. 1A). These incubation times were sufficiently short to allow attachment to spotted materials while precluding subsequent overgrowth of hyphae and mycelium onto neighboring polymer spots (Fig. 1D). Fluorescence signals from the labeled fungi were used to quantify relative attachment to the 281 homopolymers (table S1). The distribution of attachment levels across the array was broad: 3.9 and 1.1% of the polymers gave very high attachment (>1000% of the median attachment value), while 2.5 and 3.9% were strongly resistant to attachment (<10% of the median) for *C. albicans* and *B. cinerea*, respectively (Fig. 1E). Levels of attachment of the two fungi across all polymers were only weakly positively correlated, albeit significantly (Pearson correlation, *R*^2^ = 0.097, *P* < 0.0001) (Fig. 1F). Differences in the responses of the two fungi were expected given that the representative forms initiating their attachment (cells versus spores) have quite different surface properties (*22*) and the organisms themselves are phylogenetically distant among the ascomycete fungi (*23*). Nonetheless, the correlation between these fungi was closer than that we have analyzed in a similar way between the present dataset for cells of *C. albicans* and previous attachment data (*15*) for vegetative cells of the pathogenic bacteria *Escherichia coli* (*R*^2^ = 0.002), *P. aeruginosa* (*R*^2^ = 0.040), and *Staphylococcus aureus* (*R*^2^ = 0.002). Therefore, the bacterial results were very different to those obtained for fungi in this study.

Machine learning (ML) methods were used to generate predictive models for *C. albicans and B. cinerea* attachment, to assess the relationship between surface chemistry and the attachment of each fungus. Models using molecular signature descriptors (*24*) and time-of-flight secondary ion mass spectrometry (ToF-SIMS) data collected from the polymers were generated for the fungal attachment to the polymers investigated. Before modeling, sparse feature selection was used to eliminate less informative descriptors. An independent test set was used to determine the predictive power of the fungal attachment models. For both fungal species, signature descriptor (computed molecular fragments) produced the best models. Nonlinear ML models produced a small performance improvement over linear partial least square regression (figs. S1 to S3). The predictive performance of the extreme gradient boosting (XGBoost) models for *C. albicans* and *B. cinerea* attachment using signature molecular descriptors is presented in fig. S4. There was only a moderate relationship between signature molecular descriptors and observed log attachment values for *B. cinerea*, with *R*^2^ = 0.43 and RMS error (RMSE) 0.29 log fluorescence (fig. S4). A fragment descriptor relatively strongly associated with low *B. cinerea* attachment was a keto ether associated with the polymer backbone (fig. S4, C and D), although the weak predictive power of the model means that this observation should be treated with caution. For *C. albicans*, the model was stronger, with *R*^2^ = 0.70 and RMSE 0.29 log fluorescence. The molecular features that were most strongly associated with low *C. albicans* attachment were methylene nitrile C(CN) and carbonyl C(O).

Because of the noise associated with fluorescence data (figs. S3 and S4) and the relatively weak predictive power of the computational models, we adopted a reasonably liberal approach in selecting candidate materials from the screen for further interrogation, reasoning that autofluorescence, differences in polymer-spot geometry, and/or instability evident with certain polymers during the assays introduced elements of error. Therefore, for further study via scale-up, we selected 80 polymers that gave the lowest attachment for each fungus; twenty-seven of these were common to both fungi (Fig. 1F).

### Assay scale-up indicates acrylate and methacrylate polymers that are most resistant to fungal colonization

**Fig. 2:**
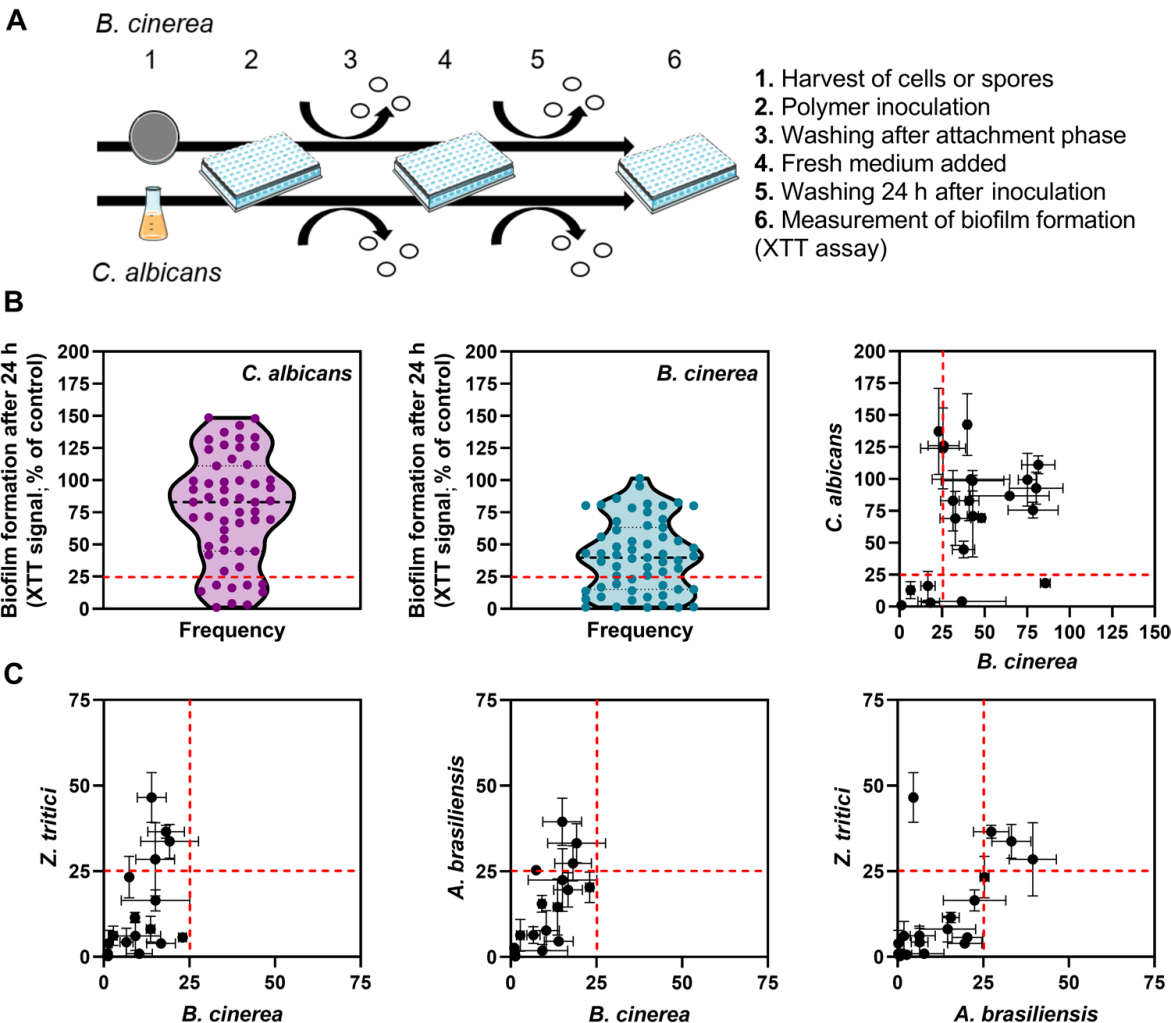
Biofilm formation on potential anti-attachment materials. Eighty polymers showing the lowest fungal attachment (from the preceding microarray-spot screen) were selected for further study. These materials were scaled up to coat the 6.4-mm-diameter wells of 96-well plates. Polymers showing surface cracking were excluded from the analysis. (**A**) Procedure for assessment of fungal biofilm formation on coated 96-well plates. (**B**) Extent of biofilm formation across the different polymers for *C. albicans* and *B. cinerea*. Materials of interest were designated as those showing <25% biofilm formation (dashed line), as compared with control microplate wells that were not coated with polymer. The full data together with names and structures of the polymers of interest are listed in tables S2 and S3. (**C**) Polymers of interest for *B. cinerea* were also tested for biofilm formation by the filamentous fungi *Z. tritici* and *A. brasiliensis.* Biofilm was again assessed after 24 hours with XTT. The *R*^2^ and *P* values for the Pearson correlations were 0.307 and 0.014 (*Z. tritici* and *B. cinerea*), 0.558 and 0.0002 (*A. brasiliensis* and *B. cinerea*), and 0.354 and 0.007 (*Z. tritici* and *A. brasiliensis*). The values are means ± SEM from at least three independent experiments.

To investigate the scalability of the polymers’ physiochemical properties and biological performance, the 80 polymers supporting the least attachment of each fungus from the microarray screen were deposited as a coating covering the 6.4-mm-diameter wells of 96-well microplates. Several of the polymers proved to exhibit surface cracking after polymerization and under the vacuum step intended to remove unreacted monomer and so were excluded from the analysis because of the presence of these additional topological features. Incubations of fungus with polymers for 24 hours were longer than in the microarray screen to allow some outgrowth and biofilm formation for a more sensitive measure of preceding attachment events; nonadherent cells or spores were removed by washing at the end of an initial attachment phase (Fig. 2A). Biofilm was detected with a metabolic XTT (tetrazolium salt, 2,3-bis[2-methyloxy-4-nitro-5-sulfophenyl]-2H-tetrazolium-5-carboxanilide) reduction assay, which eliminated the issue with autofluorescence of certain polymers. Materials of interest were designated as those supporting <25% biofilm formation compared to the control (noncoated well): <25% is equivalent to a biofilm that would result from a >95% reduction in attachment by the test fungi (fig. S5). For *C. albicans*, nine of the scaled-up polymers supported <25% biofilm formation (Fig. 2B and table S2), whereas 19 of the test polymers had such efficacy against *B. cinerea* (Fig. 2Band table S3). Of the 27 materials that were common to the two organisms in the screen and did not exhibit surface cracking (see above), only four yielded <25% biofilm formation for both organisms in the scale-up assay. In the scale-up assay, there was a similarly weak positive correlation between results for *C. albicans* cells and *B. cinerea* spores as in the preceding screen, but this was not significant in the case of the scale-up where only 23 polymers were assayed (Pearson correlation, *R*^2^ = 0.077, *P* = 0.20) (Fig. 2B). We extended the scale-up assay to test the 19 polymers giving <25% *B. cinerea* biofilm also against another major plant pathogen, *Zymoseptoria tritici*, and an environmental filamentous fungus that colonizes diverse materials, *Aspergillus brasiliensis*. There were stronger correlations between results for spores of these three fungi than with *C. albicans*, with the strongest correlation between *B. cinerea* and *A. brasiliensis* (*R*^2^ = 0.558, *P* = 0.0002) (Fig. 2C). Fifteen of the 19 polymers tested were resistant to the attachment of at least two of the three filamentous fungi (table S3).

**Fig. 3:**
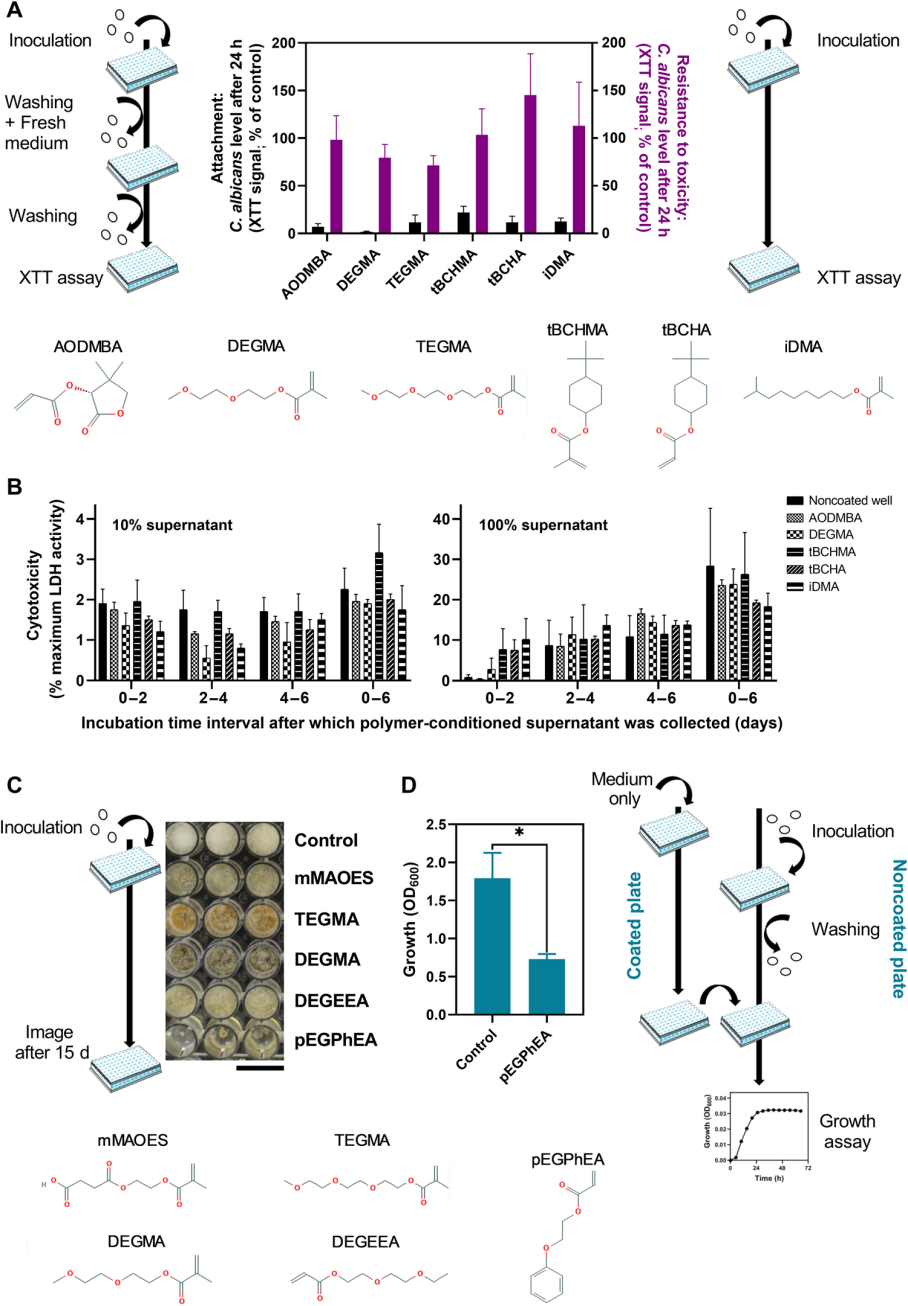
Anti-attachment versus growth inhibitory actions of selected materials. Schematics for assessing fungal attachment [(A), left] and growth inhibition [(A), right; (C); and (D)]. (**A**) Attachment and toxicity with *C. albicans*. Percentages were relative to noncoated control microplate wells. Differences between polymers were nonsignificant for either parameter [Tukey’s multiple comparisons by two-way analysis of variance (ANOVA)]. (**B**) CS from polymer-coated or uncoated wells was collected after the indicated incubation intervals [replacing with fresh Dulbecco’s modified Eagle’s medium (DMEM)] and added into 3T3 cell culture (10 or 100 μl). Lactate dehydrogenase (LDH) activity was assayed after 24 hours. Mean percentages ± SEM (*n* = 3) are relative to LDH release from lysed cells. There were no significant differences to CS from noncoated wells (Dunnett’s multiple comparisons by two-way ANOVA). TEGMA was excluded because polymer swelling complicated CS recovery. (**C**) *B. cinerea* cultivated for 15 days in polymer-coated 96-well plates. Scale bar, 1 cm. Polymerization tinted some materials. Photo credit: Cindy Vallieres, University of Nottingham. (**D**) Medium containing leached materials from pEGPhEA-coated or noncoated wells after 24 hours was transferred to wells containing *B. cinerea* spores preattached for 6 hours; then, growth was determined by OD_600_ after 24 hours (means ± SEM, *n* = 3). *P < 0.05 according to Student’s *t* test, two-tailed.

On the basis of available information on the polymers’ costs, chemistries, and toxicities, we selected nine leads deemed suitable by these criteria for further investigation: six from the *C. albicans* assay and five from *B. cinerea* (two were common to both fungi). These nine were DEGMA and TEGMA [tri(ethylene glycol) methyl ether methacrylate] (both fungi); AODMBA [(R)-α-acryloyloxy-β,β-dimethyl-γ-butyrolactone], tBCHMA (*tert*-butylcyclohexyl methacrylate), tBCHA (*tert*-butylcyclohexylacrylate), and iDMA (isodecyl methacrylate) (*C. albicans* only); and mMAOES [mono-2-(methacryloyloxy)ethyl succinate], DEGEEA [di(ethylene glycol) ethyl ether acrylate], and pEGPhEA [poly(ethylene glycol) phenyl ether acrylate] (*B. cinerea* only) (Fig. 3). As the focus was on materials that passively resisted fungal attachment, we tested for potential toxicity effects to exclude polymers that might be actively inhibiting the fungi. Here, the main technical difference with the above “anti-attachment” assays was the omission of the washing steps; all cells including any dead ones were therefore retained in the wells (Fig. 3, A and C). As there were no washing steps, the potato dextrose broth (PDB) medium could not be replaced with phosphate-buffered saline (PBS) to perform the XTT assay for toxicity in *B. cinerea*; therefore, *B. cinerea* was cultivated for 15 days on the materials in the presence of PDB and growth effects assessed visually (Fig. 3C). *C. albicans* growth was not inhibited by any of the polymers; there were no significant differences between the polymer and control (uncoated well) incubations (Fig. 3A). Furthermore, conditioned supernatant (CS) from wells coated with these polymers did not show any significantly elevated cytotoxicity to mouse 3T3 cells compared with CS from uncoated wells, either at a standard 10 or 100% CS (Fig. 3B), supporting broader biosafety. However, pEGPhEA strongly inhibited the growth of *B. cinerea* (Fig. 3C). This may have been caused by residual or leached toxic material in the medium rather than any action by the polymer. To test this hypothesis, we added PDB medium to a well containing ultraviolet (UV)–polymerized pEGPhEA and, after 24 hours, transferred this medium to wells containing *B. cinerea* spores. After 24 hours of subsequent growth, the OD_600_ (optical density at 600 nm) was 2.5-fold lower than in a control incubation (i.e., spores incubated with polymer-free medium) (Fig. 3D). This result corroborated that materials present in the medium (e.g., monomers or short oligomers leached from pEGPhEA) were toxic to *B. cinerea* growth. Given this complication, pEGPhEA was excluded from further study. In summary, the attachment data obtained for several polymers indicated that scaling up materials could alter their biological performance. However, several lead materials maintained a strong fungal anti-attachment effect, which was not attributable to toxicity.

### 3D-printed polymer forms resist colonization by *C. albicans*

**Fig. 4:**
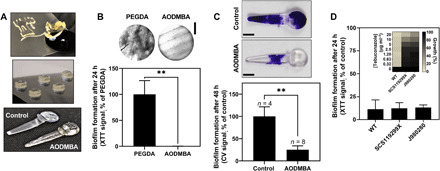
Resistance of AODMBA to *C. albicans* biofilm formation on 3D-printed polymer forms and in drug-resistant isolates. (**A**) AODMBA polymer 3D-printed into different forms including 3-mm-diameter coupons and 1.9-cm-length voice prosthesis valve flap (bottom). Photo credit: Yinfeng He, University of Nottingham. (**B**) Biofilm formation on the coupons (assayed as in Fig. 2A). PEG_575_DA (PEGDA) served as attachment positive control. Values are means ± SEM (*n* = 3). **P ≤ 0.01, unpaired *t* test. Microscopic images were taken just before XTT assay. Scale bar, 1.20 mm. (**C**) *C. albicans* biofilm, stained with crystal violet (CV), on valve flap samples 48 hours after inoculation. Biofilm that was evident with some AODMBA valve flaps detached when the form was moved or gently rinsed, unlike biofilms on commercial silicone–manufactured flaps (control). **P ≤ 0.01, unpaired *t* test. Images are representative of three or more independent attachment assays. Scale bars, 0.37 cm. Photo credit: Cindy Vallieres, University of Nottingham. (**D**) Percentage attachment to AODMBA by *C. albicans* CAF2-yCherry (WT) and azole-resistant isolates, *C. albicans* SCS119299X and J980280, relative to uncoated microplate wells. Values are means ± SEM from three independent experiments. There was no significant difference between strains according to Dunnett’s multiple comparisons by two-way ANOVA. Inset: Azole resistances of the strains.

To evaluate the potential of the polymers of interest (above) as biofilm-resistant coatings (e.g., for medical devices), we attempted to dip coat silicone coupons using polymer solutions prepared with the candidate materials. The resultant surfaces proved either to be cracked (AODMBA and tBCHMA) or to produce coatings that were both poorly adhered and prone to exhibit high levels of creep (DEGMA and TEGMA). Consequently, instead, we attempted to 3D print the target geometry directly. All the candidate monomers showed stable droplet formation during initial assessment of printability. However, during the actual printing process for 3D structures, only an AODMBA-based formulation solidified and formed stable geometries. The other candidates either remained as a tacky solid phase or collapsed during the printing, which was attributed to a low level of polymerization or low glass transition temperature (*T*_g_); analysis by differential scanning calorimetry (DSC) indicated *T*_g_ values of 86°C for AODMBA, −36°C for DEGMA, −53°C for TEGMA, and 159°C for tBCHMA. Coupons (diameter, 3 mm) manufactured with AODMBA were used initially to test the anti-attachment properties of the printed polymer (Fig. 4A). Polyethylene glycol diacrylate (PEG_575_DA) gave good *C. albicans* attachment in the previous tests (comparable to the attachment on a noncoated well) (Fig. 2B), so coupons 3D-printed with PEG_575_DA were used as attachment positive control. A ~100% reduction in *C. albicans* attachment was apparent with the AODMBA-printed coupons compared to those that were PEG_575_DA-printed (Fig. 4B). As a more commercially relevant example, next, we used AODMBA for printing valve flap forms for voice prostheses. This example was chosen because commercial silicone–manufactured valve flaps are highly susceptible in vivo to develop *C. albicans* biofilms (*21*). Our results showed that AODMBA-printed valve flaps were more resistant to fungal attachment than a standard silicone–manufactured product, with a mean 84% reduction in biofilm biomass and up to 100% reduction in some replicates (*n* = 8) (Fig. 4C). In conclusion, AODMBA was demonstrated to (i) be 3D-printable and (ii) exhibit strong anti-attachment properties that were retained in AODMBA-printed forms. As drug-resistant *C. albicans* poses particular problems for therapy, we also tested the lead material against strains resistant to azole drugs (Fig. 4D). These tests showed that anti-attachment by AODMBA is just as effective against the drug-resistant isolates as against a standard *C. albicans* strain, further supporting the potential for clinical application.

### Lead polymers can protect plant leaves from fungal infection

**Fig. 5:**
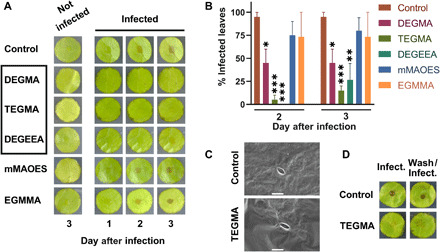
Protection against fungal infection of plant leaves. Polymers were synthesized via free-radical polymerization using a thiol chain transfer agent; see table S4 for percentage conversion, molecular weight (*M*_n_), and polydispersity (Đ). (**A**) Materials prepared at 20% (w/v) [using 20% (v/v) isopropanol solvent] were sprayed onto 1.5-cm-diameter lettuce leaf discs, before infection (right) or no infection (left) with *B. cinerea* (2500 spores per disc). Infection was examined daily up to 3 days (leaves deteriorated subsequently). The box highlights polymers giving the best protection from infection. Images are representative of three or more independent experiments, each with five leaves infected. (**B**) Percentages of infected leaves (no infection was observed at day 1). Values are means ± SEM. *P ≤ 0.05, **P ≤ 0.01, and ***P ≤ 0.001 according to Dunnett’s multiple comparisons by two-way ANOVA. EGMMA served as positive control. Growth assays confirmed no *B. cinerea* toxicity with these polymer preparations. (**C**) Scanning electron microscopy images of TEGMA-coated (bottom) or uncoated (top) leaf surfaces (×1000 magnification). Scale bars, 20 μm. ToF-SIMS analysis is in fig. S6. (**D**) TEGMA-coated leaf discs were washed with water or not before infection as in (A). Images taken at day 3, representative of five leaves infected per condition. Photo credits: Cindy Vallieres, University of Nottingham.

We then hypothesized that the lead polymers could find novel applications for protecting plant (crop) surfaces from fungal infection. To explore this possibility, first, we tested for potential plant toxicity with polymers that had given good anti-attachment against *B. cinerea* in vitro (Fig. 2B). Polymer solutions (≥85% monomer conversion; table S4) were sprayed on to lettuce leaf discs. No leaf lesions were observed up to 3 days after spraying with polymer, suggesting an absence of toxicity (leaf samples deteriorated after 3 days regardless of polymer application) (Fig. 5A, left). To test for effects on fungal infection, leaf discs treated with either polymer or solvent alone (control) were inoculated with *B. cinerea* spores. Whereas fungal lesions were evident after 2 days in all of the controls, leaves treated with either DEGEEA, DEGMA, or TEGMA were significantly resistant to *B. cinerea* infection [Fig. 5, A (middle) and B]. Fewer than 15% of TEGMA-treated leaf samples showed any sign of infection up to 3 days. In contrast, mMAOES did not confer any apparent protection as lesions appeared after 2 days: The outcome for mMAOES was similar to the untreated leaves or leaves treated with ethylene glycol methyl ether methacrylate (EGMMA), which had been selected as an attachment positive control. We confirmed that *B. cinerea* could grow in the presence of these synthesized polymer batches in vitro, so the effects on infection could not be ascribed to toxicity to the fungus. Examination of leaf surfaces coated with the best-performing polymer (TEGMA) by scanning electron microscopy (Fig. 5C) and ToF-SIMS (fig. S6) suggested good, although not complete, surface coverage. Last, we tested the resilience of TEGMA to rinsing with water. TEGMA was sprayed onto the leaf discs and air-dried as above before the leaves were rinsed three times with water and subsequently infected. After 3 days, no lesions were observed (Fig. 5D). This indicated that the anti-attachment property of the polymer conferred to the leaf surface was resilient to rinsing with water, such as may occur in the natural environment during rainfall. The presence of TEGMA after washing was confirmed by ToF-SIMS; no significant change was observed between the washed or unwashed leaf sections (fig. S6). The data were consistent with a potential for application of these materials in agriculture.

## DISCUSSION

This study identified polymer materials with the potential to stop fungal colonization by blocking attachment and demonstrates potential applications for tackling at least some of the diverse socioeconomic problems that fungi cause. Important findings that have emerged from this study include our identification of materials that block attachment of problematic microbial spores (e.g., *B. cinerea* spores), whereas previous work was restricted to vegetative bacterial cells; the lead anti-attachment materials identified are different from those described previously for bacterial pathogens, indicating substantial differences in the mechanisms of pro/anti-attachment; we identify both novel applications and routes of manufacture, including formulating the material in a form that offers protection to the surfaces of edible produce from fungal infection, and 3D-printed personalized devices that are notoriously prone to fungal deterioration.

Currently, antifungal and fungicidal agents are widely used to combat fungal pathogens, fungi causing biodeterioration, and spoilage fungi by killing them. However, with an increased incidence of fungal isolates resistant to current treatments and tightening antifungal and fungicide regulations, novel methods for fungal management are needed. Controlling fungal attachment to surfaces in a passive manner (i.e., without active killing of organisms) presents an alternative, attractive intervention at the initial step of fungal colonization. We also demonstrated absence of active killing by the polymers in cytotoxicity assays with mammalian cells, supporting the biosafety of this approach. Attachment via adhesion is a prerequisite for most adverse effects of fungi, including formation of biofilms that is an important virulence factor in microbial pathogenesis. Therefore, inhibition of attachment should be an effective target for controlling most fungi. The passive control described here could reduce the potential development of resistant organisms, as selection pressure for resistance (to anti-attachment polymers) should be considerably lower because nonresistance is not fatal and may have negligible disadvantage in many scenarios. Furthermore, resistance in this case could require organisms to gain a new function, to achieve attachment, potentially raising greater evolutionary hurdles (*25*).

For the prevention of *C. albicans* attachment on acrylic resins, chemical, or physical surface alterations such as modification of surface charge (*26*, *27*), increasing surface wettability or decreasing surface energy (*28*–*31*) previously gave lower *C. albicans* adhesion. The present study used high-throughput screening to identify polymers that are able to reduce fungal attachment from a library of more than 250 materials, the largest study of material-fungi interactions to date. High-throughput synthesis, assessment, surface characterization, and chemometrics have accelerated discovery of polymers resistant to bacterial adhesion and helped characterize chemical moieties that reduced bacterial attachment to coated medical devices in vivo (*15*). This class of materials could not have been predicted from the current understanding of bacteria-material interactions. Furthermore, variation in molecular weight between 3000 to 50,000 affected material properties but not biological anti-attachment performance (*16*). Adopting this high-throughput informatics-based approach for fungi, we identified both acrylate and methacrylate polymers that resisted fungal attachment to either biological or inert surfaces; in the latter case, the polymer described here outperforms the current market-leading silicone–manufactured material. Hydrophilic polymers have also been used in surface modification (*29*, *30*), as hydrophobic fungi preferentially adhere to hydrophobic surfaces (*32*). The lead polymers identified in this study that resisted attachment by several fungi, including TEGMA or DEGMA, were consistent with this, being broadly classified as hydrophilic with a water contact angle (WCA) of 20° to 50° (table S3). There were, however, exceptions where polymers with a WCA between 62° and 72° could still prevent fungal biofilm. The lead polymers resisting attachment only of *C. albicans* were more hydrophobic, with WCAs of 62° to 96°. Thus, the ability of these polymers to prevent fungal biofilm formation indicates that hydrophilicity alone is not sufficient to predict fungal attachment to a particular material.

In this study, we were able to use AODMBA for inkjet-based 3D printing to demonstrate the capability to manufacture bespoke fungal attachment–resistant devices. The anti-attachment properties of AODMBA were retained after printing, including in a printed medical device component (valve flap for a voice prosthesis). One of the advantages of manufacturing these parts with polymer rather than coating the polymer onto target devices is that the object is homogenous, and thus, it is less likely that there will be exposure of regions of (potentially attachment-prone) native surface. The AODMBA-printed valve flaps showed >80% reduction of biofilm formation compared with a standard silicone–manufactured product. However, AODMBA forms a hard, glassy polymer that would therefore be, in its homopolymer form, too inflexible for valve flap applications. This is analogous to the antibacterial polymer EGDPEA development, in which the homopolymer was also not suitable to produce a viable catheter coating (*16*); rather, an optimized copolymer had to be developed. Similarly, to develop a commercially viable coating, the mechanical properties of that material were improved by copolymerization with a comonomer (DEGMA) that has a lower *T*_g_. In practice, the *T*_g_ values that were exhibited by poly(EGDPEA-*co*-DEGMA) polymers, synthesized in various different comonomer ratios, were used as a high-throughput screening guide to predict the copolymer compositions that should match the flexibility of the commercial catheter material. Pin printing assays using those copolymers with acceptable *T*_g_ confirmed that they still retained the bacterial attachment resistance (*16*). Similar optimization could be an aim of future work to improve the mechanical properties of AODMBA-based forms. This could include copolymerization with lower *T*_g_ comonomers, and the screening data presented here suggest a number of potentially suitable candidate comonomers that themselves exhibit some anti-attachment properties. By observing the latter point, any loss of anti-attachment in copolymer materials should be minimized. Design guidance for materials may, of course, need to be tailored for different fungal attachment scenarios. The present work is a key step toward deriving a set of molecular design rules to define even more relevant molecular structures to be incorporated in materials to improve performance. Our analyses showed that surface chemistry is not a very strong differentiator for fungal attachment, suggesting that material properties will play a more significant part in performance compared with the bacterial work.

In agriculture, polymer materials have found applications for improving physical properties of soil and as adjuvants in polymeric biocide and herbicide formulations. These latter are controlled-release formulations designed to reduce the possible side effects accompanying the overuse of biologically active agents. However, the passive application proposed in the current study is a novel potential replacement for active agents in formulations. These materials were effective against *B. cinerea* and *Z. tritici*, two major crop pathogens. Furthermore, three of four selected polymers conferred plant protection against *B. cinerea* infection. TEGMA, the best-performing polymer, showed resistance to the attachment of all four fungi used in this study, suggesting a broad spectrum of action of this methacrylate material. Broad-spectrum agents are particularly valued in common antimicrobial applications, including crop protection.

In conclusion, this work identified a panel of polymers that are resistant to fungal attachment and therefore reduce fungal biofilm formation and infection. Besides the therapeutic and crop protection potential, such acrylate and methacrylate polymers have wider applications, as exemplified by their effect on the attachment of *A. brasiliensis*, known to colonize synthetic products and materials. This study is an important first step toward the targeted design of novel materials tailored for different antifungal applications.

## MATERIALS AND METHODS

### Polymer array synthesis

Polymer microarrays were prepared using a modified version of the previously described procedure (*33*). Polymer microarrays were printed using an XYZ3200 dispensing station (BioDot) using quilled steel pins (Arrayit, 946MP6B). Printing was carried out under an argon atmosphere maintaining O_2_ < 2000 parts per million (ppm), 25°C, and 30 to 35% relative humidity. Diluted polymerization solutions were composed of monomer [50% (v/v) for oils and 50% (w/v) for solids] in *N*,*N′-*dimethylformamide, 1:1 *N*,*N′-*dimethylformamide:water or 1:1 *N*,*N′-*dimethylformamide:toluene depending on solubility. The photoinitiator 2,2-dimethoxy-2-phenyl acetophenone [1% (w/v)] was added to all solutions. A total of three replicates were printed on each slide, each replicate comprising 281 different polymers (table S1). Monomers were purchased from Sigma-Aldrich, Scientific Polymers, Acros Organics, or Polysciences and were used as received. Spacing between the printed spots in each row was 1500 μm in the *x* axis, with an alternating +750-μm/−750-μm offset in the *x* axis between each row and a 750-μm spacing between each row in the *y* axis. After printing was completed, arrays were dried in a Heraeus vacuum oven (35°C, 0.3 mbar) for 7 days.

### High-throughput surface characterization

ToF-SIMS measurements were conducted using a ToF-SIMS IV (IONTOF GmbH) instrument operated using a 25-kV Bi_3_^+^ primary ion source exhibiting a pulsed target current of ∼1 pA. Samples were scanned at a pixel density of 100 pixels/mm, with eight shots per pixel over a given area. The analysis area was 20,000 μm by 20,000 μm. An ion dose of 2.45 × 10^11^ ions/cm^2^ was applied to each sample area, ensuring that static conditions were maintained throughout. Both positive and negative secondary ion spectra were collected (mass resolution of >7000 at mass/charge ratio of 29). Owing to the nonconductive nature of the samples, a low-energy (20 eV) electron flood gun was applied to provide charge compensation. WCA was measured as described previously (*34*).

### Computational modeling

The replicate fungal fluorescence values for each of the polymers screened (three replicates for *B. cinerea* and six for *C. albicans*) were averaged, and the SDs were calculated. As the fluorescence values spanned a wide range, the log of the fluorescence values was used as the dependent variable in the computational models, as is common practice for quantitative structure-activity relationship modeling. Polymers with low signal-to-noise ratio (<1.5) were excluded from the *B. cinerea* (173 polymers) and *C. albicans* (197 polymers) attachment datasets. For modeling, least absolute shrinkage and selection operator (LASSO) was used to select sparse subsets of features from larger pools of possibilities in a context-dependent manner.

Partial least square regression was conducted using MATLAB R2018a 9.4.0.813654. ToF-SIMS positive and negative data were concatenated into a single data matrix to be used as the *X* variables for the model. *X* variables were mean-centered and variance-scaled before analysis. Data were randomly split into training and test sets (3:1) to validate the model produced. The number of latent variables used in the model was selected on the basis of a minimum in the RMSE of cross validation. Three latent variables were selected for models for each fungal species.

XGBoost regression ML, a robust nonlinear ML method (*35*), was used to generate models relating chemical features to fluorescence (and, therefore, attachment). The chemical features used to train the models were of two types: signature molecular descriptors (*36*, *37*) generated by computationally fragmenting molecules and ToF-SIMS ion peaks derived from actual molecular fragmentation by probe ions. Although independent test sets are the best way of assessing the predacity of ML models, because of the high variability and noise present in the datasets, leave-one-out (LOO) cross-validation was used for this purpose.

The XGBoost algorithm (version 0.22) (*35*) with default parameters was used to generate the models, and LOO cross validation was implemented using the package LeaveOneOut from sklearn.model_selection (both codes were implemented in Python 3.7). LASSO feature selection was implemented in MATLAB R2017a using the lassoglm function, selecting the features that provide the minimum value for the squared error for the lambda parameter. Their rank in importance is given by the XGBoost descriptor importance parameter, which provides a score indicating how useful each descriptor was in constructing the boosted decision trees within the model, using Gini as performance measure. This importance was calculated for each descriptor and averaged across the multiple trees, allowing attributes to be ranked and compared to each other.

### Free-radical polymerization scale-up for performance validation, inkjet 3D printing, and leaf coating

#### *Polymerization method for biological performance validation*

The synthesis of selected compounds was upscaled to allow the validation of the biological performance observed in the pin printing assays. This was achieved by coating the 6.4-mm-diameter wells of 96-well plates. Plates were prepared by adding 50 μl of monomer solution into each well. Polymerization was initiated by addition of 2,2-dimethoxy-2-phenylacetophenone (Sigma-Aldrich) to a final concentration of 1% (w/v). Samples were irradiated with UV (Blak-Ray XX-15L UV Bench Lamp; 230 V, ~50 Hz, 15 W, 365 nm) for 1 hour with O_2_ < 2000 ppm. The samples were dried at <50 mtorr for 7 days. Wells were then washed briefly with isopropanol and left for 2 days at 37°C in distilled water. Plates were then washed briefly with isopropanol and distilled water and air-dried before irradiation with UV for 20 min to sterilize the samples.

#### *Polymerization method for validation of inkjet 3D printing performance*

Exploring the potential printability of a monomer for inkjet-based 3D printing requires consideration of several key factors including viscosity, surface tension, printing conditions, etc. Following existing methods for the efficient formulation development of inkjet-based 3D printing inks (*38*–*41*), candidate monomers that were suitable for the inkjet 3D printing process were identified, and then, associated ink formulations were prepared by dissolving 1% (w/v) 2,2-dimethoxy-2-phenylacetophenone (Sigma-Aldrich) into 5 ml of the candidate monomer. The mixture was stirred at 800 rpm at room temperature until the initiator was fully dissolved. The ink was then purged with nitrogen gas for 15 min and filtered through a 5-μm nylon syringe filter. The final ink formulation was left at 4°C overnight to degas. A Dimatix DMP-2830 material printer was used for printing, equipped with a 10-pl cartridge containing 16 nozzles, each with a square cross section with a side length of 21 μm. The jetting voltage and waveform were adjusted until stable droplet formation was achieved. A 365-nm UV light-emitting diode (LED) unit (800 mW/cm^2^) was used for in-line swath-by-swath ink curing after deposition. The whole printing process was carried out in a nitrogen environment, where the oxygen level was 0.2 ± 0.05%.

#### *Polymerization method for leaf coating*

For investigating the fungal infection of polymer-coated plant leaves, polymerization of the materials identified as candidates for resistance to fungal infection was performed by free-radical polymerization using a thiol chain transfer agent to limit the molecular weight of the final material and ensure that it was processable. Candidate monomers were dissolved in cyclohexanone (Acros Organics; 1:3, v/v), and the chain transfer agent [1-dodecanethiol (Acros Organics), 5% (mol/mol) with respect to the monomer] and initiator [2′-azobis(2-methylpropionitrile; Sigma-Aldrich; 0.5% (w/w)] were added. Argon was purged into the mixture for 30 min to remove oxygen, before holding at 75°C for 24 hours. Isolation of the polymer was achieved by precipitation into an excess of either (i) heptane (Fisher Scientific; DEGEEA, DEGMA, EGMMA, and TEGMA) or (ii) chloroform (Fisher Scientific; mMAOES). The nonsolvent to reaction mixture ratio used for the precipitations was 5:1 (v/v). Precipitated materials were collected in vials and incubated in a vacuum oven for at least 24 hours before use. Nuclear magnetic resonance (NMR) spectroscopic analysis was performed with the crude polymerization solution to determine polymer conversion and on the final precipitate to assess purity. To evaluate the molecular weight of the materials, purified samples were dissolved in high-performance liquid chromatography (HPLC)–grade tetrahydrofuran (THF) for analysis by gel permeation chromatography (GPC).

### Differential scanning calorimetry

Polymer thermal properties were investigated by DSC (Q2000, TA Instruments, Leatherhead, UK), at a heating rate of 10°C min^−1^. Data analysis was done with TRIOS software (version 4.4.0.40883). Pans with holed lids (TA Instruments, Brussels, Belgium) were used for sample analysis, with empty pans as the reference. Glass transitions were determined by performing two heating/cooling cycles between −90° and 200°C.

### ^1^H NMR spectroscopy

^1^H NMR spectra were recorded at 25°C using a Bruker DPX-300 spectrometer (400 MHz). Chemical shifts were recorded in δH (in ppm). Samples were dissolved either in deuterated chloroform (DEGEEA, DEGMA, EGMMA, and TEGMA) or in deuterated acetone (mMAOES) to which chemical shifts were referenced (residual chloroform at 7.26 ppm and residual acetone at 2.05 ppm).

### Gel permeation chromatography

GPC analysis was performed using an Agilent 1260 Infinity instrument equipped with a double detector in the light scattering configuration. Two mixed columns at 25°C were used, using THF as the mobile phase at a flow rate of 1 ml min^−1^. GPC samples were prepared in HPLC-grade THF and filtered before injection to the GPC system. Analysis was carried out using Astra software. The molecular weight (number average, *M*_n_) and polydispersity (Ð) were calculated, with reference to a calibration curve created using commercially purchased poly(methyl methacrylate) standards.

### Microscopy

Optical microscopy of polymer microarray slides was with a GX microscope (GXM-L3201 LED). AFM measurements of polymer microarrays were acquired as previously reported (*42*) using a Bruker FastScan Icon AFM with Bruker TAP150A spring (5 N/m) constant tips. Polymer spots were analyzed in batches of 100, with polystyrene controls taken at 15-measurement intervals to ensure that tips were not damaged during a run. Images were analyzed using Gwyddion 2.55 software. Scanning electron microscopy imaging of polymer-coated lettuce leaves was conducted with a JEOL JSM-6490LV. Samples were gold-coated before imaging using a Polaron SC7640 sputter coater.

### Fungal growth conditions

The main fungal strains used in this study were the yeast *C. albicans* CAF2-yCherry [provided by R. Wheeler, University of Maine, USA; (*43*)] and the filamentous fungi *B. cinerea* SAR109940, *Z. tritici* K4418, and *A. brasiliensis* CBS 246.65. Azole-resistant isolates of *C. albicans* (SCS119299X and J980280) were provided by C. Munro and D. Maccullum, University of Aberdeen, UK. *C. albicans* was maintained and grown in YPD (yeast extract, peptone, and dextrose) medium [2% peptone (Oxoid, Basingstoke, UK), 1% yeast extract (Oxoid), and 2% d-glucose] (*44*). Where necessary, medium was solidified with 2% (w/v) agar (Sigma-Aldrich, UK). The filamentous fungi were routinely maintained and grown on potato dextrose agar (PDA; Oxoid) or PDB (Sigma-Aldrich, UK).

### Polymer microarray screening for fungal attachment

Before testing against fungi, the microarray slides were washed by immersion in distilled water for 10 min, air-dried, and UV-sterilized. For screening with *C. albicans* (yCherry-tagged), single colonies were used to inoculate YPD broth cultures in Erlenmeyer flasks and incubated at 37°C with orbital shaking at 150 revolutions min^−1^. Overnight cultures were washed twice in RPMI 1640 (Sigma-Aldrich) and adjusted to OD_600_ ~ 10. Microarray slides were incubated statically at 37°C for 2 hours with 15 ml of the cell suspension. For tests with *B. cinerea*, spores were harvested from 7-day-old PDA plates, washed twice in PDB medium, and resuspended in PDB at a concentration of 2 × 10^7^ spores ml^−1^. As with *C. albicans*, microarray slides were incubated statically with 15 ml of the cell suspension but at room temperature for 6 hours and stained for 10 min with 0.5% Congo red. As controls, slides were also incubated with noninoculated medium. After the period of attachment, the slides were removed and washed three times with 15 ml of PBS at room temperature. After rinsing with distilled water to remove salts then air drying, fluorescence images from the slides were captured using either a GenePix Autoloader 4200AL (*C. albicans*; Molecular Devices, USA) or 4000B (*B. cinerea*; Molecular Devices, USA) scanner, with a 635-nm red laser and red emission filter. The total fluorescence signal from each polymer spot was determined using GenePix Pro 6 software (Molecular Devices, USA). The fluorescence signal attributable to fungal attachment to each polymer was determined by subtracting the fluorescence signal in the medium-only control incubation from that in the incubation with fungus. For polymers where the fluorescence was below the limit of detection, fluorescence was recorded as zero, as discussed in (*15*). Fungal attachment to each polymer is expressed as a percentage relative to the median value (equal to 100%) across all polymers for each fungus.

### Fungal biofilm assessment

Biofilm metabolic activity was measured by the XTT (Sigma-Aldrich) reduction assay. For *C. albicans*, single colonies were used to inoculate YPD broth cultures in Erlenmeyer flasks and incubated overnight at 37°C with orbital shaking at 150 revolutions min^−1^. Cultures were washed twice in RPMI 1640 and diluted to 125,000 cells ml^−1^. Aliquots (100 μl) of the cell suspension were transferred to 96-well microtiter plates (Greiner Bio-One, Stonehouse, UK), either coated with the polymers of interest or containing coupons 3D-printed with polymer as described above and then incubated statically for 2 hours. Similarly, 100 μl of fungal spores (2.5 × 10^6^ spores ml^−1^ in PDB) from 7-day-old PDA plates were transferred to coated 96-well plates for 6 hours at room temperature. In all cases, coupons were subsequently transferred to fresh 96-well plates. Nonadherent cells or spores were removed by three gentle washes with PBS; then, 100 μl of fresh medium was added to each well, and plates were incubated at 37°C up to 24 hours after inoculation. Coupons were again transferred to fresh plates. The wells were washed three times with PBS, and the XTT reaction was initiated by adding XTT and menadione to RPMI (for *C. albicans*) to final concentrations of 210 μg ml^−1^ and 4.0 μM, respectively, or to PBS (for *B. cinerea*) to final concentrations of 400 μg ml^−1^ and 25 μM (final volume per well, 200 μl; PBS was used instead of PDB, as the XTT reaction does not work in PDB medium). After 2 and 6 hours, respectively, 100 μl of the reaction solutions was transferred to fresh 96-well plates, and the absorbance at 490 nm was measured using a BioTek EL800 microplate spectrophotometer. To assess the impact of the polymers on fungal growth, washing steps were omitted as presented in Fig. 3. Contrary to RPMI and as mentioned above, the XTT reaction cannot be performed in PDB medium, and fungi are not able to grow in PBS. Therefore, *B. cinerea* was cultivated for 15 days on the polymers in the presence of PDB, and growth effects were assessed visually.

Biofilm formation was also assessed on prosthesis valve flaps, either printed (above) or commercial manufactures from silicone (provided by Atos Medical; raw material is Silastic Q7-4735, Dow Corning). The latter was used as the control material. The materials were immersed in the presence of 1 × 10^6^ cells in RPMI 1640 (final volume, 1 ml) in 12-well plates (Greiner Bio-One). After 2 hours of static incubation at 37°C, valve flaps were transferred to new plates and washed three times with PBS to remove nonadherent cells. Fresh RPMI 1640 was added. After 46 hours at 37°C with orbital shaking at 100 revolutions min^−1^, RPMI 1640 was removed and biofilm stained with 0.5% (w/v) crystal violet for 1 min. The valve flaps were washed three times with PBS to remove nonadherent biofilm and excess stain before image capture. For quantification, the crystal violet was dissolved with 1 ml of ethanol and 100 μl of the reaction was transferred to 96-well plates. Absorbances at 600 nm were measured using a BioTek EL800 microplate spectrophotometer.

### Cytotoxicity assessment

Immortalized National Institutes of Health 3T3 mouse embryonic fibroblast cells (passage 25) were cultured in Dulbecco’s modified Eagle’s medium (DMEM; Sigma-Aldrich) supplemented with 10% fetal bovine serum, 2 mM l-glutamine (Sigma-Aldrich), and penicillin (100 U ml^−1^ ) + streptomycin (100 μg ml^−1^) (Gibco) in 75-cm^2^ cell-culture flasks (Greiner Bio-One) at 5% CO_2_ and 37°C. Cells were detached with trypsin/EDTA (Sigma-Aldrich) and washed with DMEM before dispensing 100 μl of cell suspension at 5000 cells per well in a 96-well plate. After 24 hours, either 10 or 100 μl of the medium was replaced with CS (below) and incubated for a further 24 hours. For CS, 200 μl of supplemented DMEM (above) was added to polymer-coated or uncoated wells of plates (prepared as described in the “Polymerization method for biological performance validation” section) and collected at intervals, with recovered CS replaced by fresh medium for a subsequent interval. Toxicity of CS to the 3T3 cells was according to release of lactate dehydrogenase (LDH) into the cell culture medium, determined with the CyQUANT LDH Cytotoxicity Assay Kit (Thermo Fisher Scientific) according to the manufacturer’s protocol. Cytotoxicity was calculated relative to controls for spontaneous LDH activity (cells grown in fresh medium) and maximum activity (cells treated with CyQUANT lysis buffer).

### Fungal infection of plant leaves

Polymer solutions [20% (w/v), prepared using 20% (v/v) isopropanol as solvent] were sprayed onto 1.5-cm-diameter leaf discs prepared from fresh lettuce. Discs were placed onto water agar [sterile distilled water, 2% (w/v) agar] in square Petri plates (Greiner) and then incubated at room temperature for up to 3 days. To measure resilience of coated polymer to rinses with water, some lettuce leaf discs were washed by submersion in water. Spores of *B. cinerea* were harvested from 7-day-old PDA plates, washed twice with PDB, and adjusted in PDB to a concentration of 5 × 10^5^ spores ml^−1^. Once dried, leaf discs were infected with *B. cinerea* by aliquoting 5 μl of spore suspension to the middle of the discs (2500 spores per leaf disc). Images were captured every day up to 3 days after infection to assess lesions. To assess potential toxicity of polymers to the plant material, leaf discs were sprayed with the polymers but not infected with *B. cinerea*.

### Statistical analysis

Statistical analyses were carried out by Student’s *t* test, Pearson correlation, or two-way analysis of variance (ANOVA), using Prism software, from a minimum of three independent replicate values. Regression models used for the computational modeling are detailed above and in the Supplementary Materials.

## Supplementary Material

aba6574_SM.pdf

## SUPPLEMENTARY MATERIALS

Supplementary material for this article is available at http://advances.sciencemag.org/cgi/content/full/6/23/eaba6574/DC1

## Appendix

View/request a protocol for this paper from *Bio-protocol*.
