# Supplementary material for: Discovery of (meth)acrylate polymers that resist colonization by fungi associated with pathogenesis and biodeterioration
Source: Sci Adv. 2020 Jun 5;6(23):eaba6574. doi: 10.1126/sciadv.aba6574 (PMC7274803; doi:10.1126/sciadv.aba6574)
Supplement: aba6574_SM.pdf [file aba6574_SM.pdf]

Science Advances
AAAS

advances.sciencemag.org/cgi/content/full/6/23/eaba6574/DC1

Supplementary Materials for

Discovery of (meth)acrylate polymers that resist colonization by fungi associated with pathogenesis and biodeterioration

Cindy Vallieres, Andrew L. Hook, Yinfeng He, Valentina Cuzzucoli Crucitti, Grazziela Figueredo, Catheryn R. Davies, Laurence Burroughs, David A. Winkler, Ricky D. Wildman, Derek J. Irvine, Morgan R. Alexander*, Simon V. Avery*

*Corresponding author. Email: simon.avery@nottingham.ac.uk (S.V.A.); morgan.alexander@nottingham.ac.uk (M.R.A.)

Published 5 June 2020, *Sci. Adv.* **6**, eaba6574 (2020)
DOI: 10.1126/sciadv.aba6574

This PDF file includes:

Tables S1 to S4
Figs. S1 to S6

SUPPLEMENTARY MATERIALS

Table S1. Polymer identities and attachment data from the polymer microarray screen.

**Acronym**	**Attachment[a]**		**Full chemical name**
	Botrytis	*Candida*	
**13BDDA**	98.0	100.0	Butanediol-1,3 diacrylate
**13BDDMA**	36.7	105.9	1,3-Butanediol dimethacrylate, 98%
**14BDDMA**	73.5	80.6	1,4-Butanediol dimethacrylate
**AA**	238.8	248.8	Allyl acrylate
**AAcAm**	63.3	92.4	Diacetone acrylamide
**AAm**	0.0	206.5	Acrylamide
**AcAPAm**	247.0	59.4	N-[2-(Acryloylamino)phenyl]acrylamide
**AEMA.C**	477.6	1778.1	2-Aminoethyl methacrylate hydrochloride,
**AEMAm.C**	496.0	1948.7	N-(2-aminoethyl) methacrylamide hydrochloride
**AMA**	198.0	152.2	Allyl methacrylate
**AnMA**	128.6	249.8	Anthracenylmethylacrylate
**AODMBA**	14.3	36.6	(R)-α-Acryloyloxy-β,β-dimethyl-γ-butyrolactone
**AOHPMA**	14.3	11.4	Acryloyloxy-2-hydroxypropyl methacrylate
**APMAm.C**	185.8	2136.7	N-(3-Aminopropyl)methacrylamide hydrochloride
**BA**	210.2	372.7	Butyl acrylate
**BAC**	95.9	70.1	N,N'-Bis(acryloyl)cystamine
**BACOEA**	379.7	80.1	Butylamino carbonyl oxy ethyl acrylate
**BAGDA**	4.1	45.2	Bisphenol A glycerolate diacrylate
**BAPA**	122.5	130.2	1,4-Bis(acryloyl)piperazine
**BAPODA**	149.0	81.3	Bisphenol A propoxylate diacrylate
**BDDA**	91.9	159.0	Butanediol diacrylate
**BFEODA**	149.0	83.1	Bisphenol F ethoxylate diacrylate
**BHMA**	51.0	37.0	Benzhydryl methacrylate
**BHMOPhP**	104.1	62.3	2,2-Bis[4-(2-hydroxy-3-methacryloxypropoxy)phenyl]propane
**BMA**	165.3	441.1	Butyl methacrylate
**BMAM**	193.9	19.2	N-Benzylmethacrylamide
**BMAOEP**	342.9	477.7	Bis[2-(methacryloyloxy)ethyl] phosphate
**BMENBC**	118.4	19.6	Bis(2-methacryloxyethyl) N,N'-1,9-nonylene biscarbamate
**BnA**	49.0	152.8	Benzyl acrylate
**BnMA**	32.7	96.8	Benzyl methacrylate
**BnPA**	38.8	90.0	Benzyl 2-n-propyl acrylate
**BOEMA**	24.5	110.6	Butoxyethyl methacrylate
**BOMAm**	318.4	76.6	N-(Butoxymethyl)acrylamide
**BPAPGDA**	149.0	142.9	Bisphenol A propoxylate glycerolate diacrylate
**BPDMA**	71.4	0.0	Bisphenol A dimethacrylate
**BPEODA**	67.4	8.9	Bisphenol A ethoxylate diacrylate
**BTHPhMA**	187.8	69.1	Benzotriazol-2-yl)-4-hydroxyphenyl]ethyl methacrylate
**BzHPEA**	130.6	21.4	Benzoyl-3-hydroxy-phenoxy)ethyl acrylate
**CEA**	244.9	21.1	Carboxyethyl acrylate
**CHA**	165.3	102.0	Cyclohexyl acrylate
**CHMA**	55.1	125.2	Cyclohexyl methacrylate
**CHPMA**	1226.8	44.1	Chloro-2-hydroxy-propyl methacrylate
**CMAOE**	49.0	75.7	Caprolactone 2-(methacryloyloxy)ethyl ester
**CNEA**	36.7	48.0	Cyanoethyl acrylate
**COEA**	112.3	48.4	2-Cinnamoyloxyethyl acrylate

**CzEA**	1808.5	53.3	Carbazol-9-yl ethyl acrylate
**DAAM**	59.2	65.3	N,N-Diallylacrylamide
**DDDMA**	49.0	162.8	1,10-Decanediol dimethacrylate
**DEAEA**	18.4	3005.2	Diethylamino ethyl acrylate
**DEAEMA**	126.6	1399.8	Diethylaminoethyl methacrylate
**DEGDA**	55.1	28.7	Di(ethylene glycol) diacrylate
**DEGDMA**	51.0	20.8	Diethylene glycol dimethacrylate
**DEGEEA**	49.0	102.8	Di(ethylene glycol) ethyl ether acrylate
**DEGEHA**	71.4	110.3	Di(ethylene glycol) 2-ethylhexyl ether acrylate
**DEGMA**	22.5	33.4	Di(ethylene glycol) methyl ether methacrylate
**DFFMOA**	228.6	78.8	Dodecafluoro-7-(trifluoromethyl)-octyl acrylate
**DFHA**	142.9	79.3	Dodecafluoroheptyl acrylate
**DFHNMA**	110.2	298.2	Dodecafluoro-2-hydroxy-8-(trifluoromethyl)nonyl methacrylate
**DHEBAM**	387.8	696.5	N,N'-(1,2-Dihydroxyethylene)bisacrylamide
**DiPEMA**	40.8	154.7	2-Diisopropylaminoethyl methacrylate
**DMA**	128.6	240.9	Decyl methacrylate
**DMAEA**	102.1	98.1	Dimethylamino-ethyl acrylate
**DMAEMA**	536.8	3276.6	Dimethylamino-ethyl methacrylate
**DMAm**	2.0	150.5	N,N'-Dimethylacrylamide
**DMAPA**	559.3	7964.3	Dimethylamino-propyl acrylate
**DMEMAm**	1061.4	1878.9	N-[2-(N,N-Dimethylamino)ethyl]methacrylamide
**DMMAm**	49.0	44.6	N,N-Dimethylmethacrylamide
**DMPAm**	553.2	4083.7	N-[3-(Dimethylamino)propyl]acrylamide
**DMPMAm**	236.8	747.6	N-[3-(Dimethylamino)propyl]methacrylamide
**DOAm**	46.9	82.6	Disperse Orange 3 acrylamide
**DPEPHA**	167.4	33.0	Dipentaerythritol penta/hexa-acrylate
**DRA**	169.4	1042.7	Disperse red 1 acrylate
**DVAd**	104.1	77.3	Divinyl Adipate
**DVSeb**	85.7	306.3	Divinyl sebacate
**DYA**	185.8	291.9	Disperse yellow 7 acrylate
**E3GDA**	57.2	167.9	Triethylene glycol diacrylate
**EA**	124.5	98.3	Ethyl acrylate
**EaNIA**	100.0	180.1	Ethyl trans-a-cyano-3-indole-acrylate
**EBAM**	357.2	432.7	N,N'-Ethylenebisacrylamide
**EbCNA**	69.4	30.7	Ethyl-cis-B-cyano-acrylate
**ECNTA**	310.3	408.9	Ethyl-2-cyano-3-(2-thienyl)acrylate
**EEA**	140.8	114.6	Ethyl 2-ethylacrylate
**EEMA**	44.9	94.3	Ethoxyethyl methacrylate
**EG3DMA**	49.0	37.5	Tri(ethylene glycol) dimethacrylate
**EG4DMA**	79.6	44.1	Tetraethylene glycol dimethacrylate
**EGDA**	53.1	100.6	Ethylene glycol diacrylate
**EGDCMA**	57.2	106.2	Ethylene glycol dicyclopentenyl ether methacrylate
**EGDMA**	49.0	67.7	Ethylene glycol dimethacrylate
**EGDPEA**	51.0	266.7	Ethylene glycol dicyclopentenyl ether acrylate
**EGMEA**	42.9	61.1	Ethylene glycol methyl ether acrylate
**EGMMA**	44.9	64.0	Ethylene glycol methyl ether methacrylate
**EGPEA**	65.3	101.6	Ethylene glycol phenyl ether acrylate
**EGPhMA**	75.5	36.2	Ethylene glycol phenyl ether methacrylate
**EHA**	202.1	179.3	Ethylhexyl acrylate
**EHMA**	238.8	66.9	Ethylhexyl methacrylate
**EMA**	167.4	151.8	Ethyl methacrylate
**EOEA**	51.0	73.7	Ethoxyethyl acrylate
**EPA**	100.0	221.1	Ethyl 2-propylacrylate
**ExA**	181.7	242.2	Epoxidized acrylate

**F6BA**	63.3	57.0	Hexafluorobutyl acrylate
**F6BMA**	46.9	46.3	Hexafluorobutyl methacrylate
**F7BA**	55.1	356.6	Heptafluorobutyl acrylate
**F7BMA**	100.0	117.5	Heptafluorobutyl methacrylate
**FMHPNMA**	85.7	15.4	Trifluoro-2′-(trifluoromethyl)-2′-hydroxy)propyl]-3-norbornyl methacrylate
**FuMA**	10.2	62.9	Furfuryl methacrylate
**FuMA/tBAEMA**	253.1	484.0	
**GA**	683.8	327.4	Glycidyl acrylate
**GDGDA**	342.9	135.2	Glycerol 1,3-diglycerolate diacrylate
**GDMA**	75.5	53.0	Glycerol dimethacrylate
**GMA**	228.6	475.9	Glycidyl methacrylate
**GMMA**	455.2	268.5	Glycerol monomethacrylate
**GPOTA**	42.9	63.1	Glycerol propoxylate triacrylate
**HA**	85.7	116.5	Hexyl acrylate
**HBA**	132.7	66.1	Hydroxybutyl acrylate
**HBMA**	16.3	86.7	Hydroxybutyl methacrylate
**HBOPBA**	98.0	325.3	Hexanediylbis[oxy(2-hydroxy-3,1-propanediyl)] bisacrylate
**HDDMA**	42.9	44.2	1,6-Hexanediol dimethacrylate,
**HDFDA**	220.5	210.4	Heptadecafluorodecyl acrylate
**HDFDMA**	81.6	79.9	Heptadecafluorodecyl methacrylate
**HDFHUA**	165.3	345.4	Heptadecafluoro-2-hydroxyundecyl acrylate
**HDMA**	253.1	815.7	1-Hexadecyl methacrylate
**HDMPDA**	116.4	31.9	Hydroxy-2,2-dimethylpropyl 3-hydroxy-2,2-dimethylpropionate diacrylate
**HEA**	102.1	130.8	Hydroxyethyl acrylate
**HEAm**	65.3	205.6	N-Hydroxyethyl acrylamide
**HEODA**	65.3	122.1	Hexanediol ethoxylate diacrylate
**HEODA/EEMA**	124.5	76.3	
**HfCEA**	34.7	789.3	Hafnium carboxyethyl acrylate
**HFDA**	177.6	363.5	Heneicosafluorododecyl acrylate
**HFHUMA**	120.4	322.4	Hexadecafluoro-2-hydroxy-10-(trifluoromethyl)undecyl methacrylate
**HFiPA**	83.7	37.6	Hexafluoroisopropyl acrylate
**HFiPMA**	110.2	75.3	Hexafluoroisopropyl methacrylate
**HFPDA**	155.1	52.1	Hexafluoropent-1,5-diyl diacrylate
**HMA**	273.5	67.0	Hexyl methacrylate
**HMAm**	91.9	65.6	N-(Hydroxymethyl)acrylamide
**HMBAM**	528.7	134.3	N,N′-Hexamethylenebisacrylamide
**HMBMAm**	281.7	107.2	N,N'-Hexamethylenebis(methacrylamide)
**HPA**	51.0	79.2	Hydroxypropyl acrylate
**HPhMA**	85.7	726.8	N-(4-Hydroxyphenyl)methacrylamide
**HPhOPA**	181.7	0.0	Hydroxy-3-phenoxypropyl acrylate
**HPHPBAH**	59.2	117.6	Hydroxypivalyl hydroxypivalate bis[6-(acryloyloxy)hexanoate]
**HPMA**	130.6	250.2	Hydroxypropyl methacrylate
**HPMAm**	132.7	591.9	N-(2-Hydroxypropyl)methacrylamide
**HPMAP**	112.3	147.3	Hydroxypropyl 2-(methacryloyloxy)ethyl phthalate
**HTFDA**	200.0	277.6	Hexadecafluoro-9-(trifluoromethyl)decyl acrylate
**iBA**	330.7	139.8	Isobutyl acrylate
**IBESMA**	102.1	82.6	1,7,7-trimethylbicyclo[2.2.1]heptan-2-yl 6-(methacryloyloxy)-4-oxohexanoate
**iBMA**	57.2	85.8	Isobornyl methacrylate
**iBMA/tBAEMA**	193.9	975.9	
**iBOA**	61.2	73.7	Isobornyl acrylate
**iBOMAm**	175.5	140.9	N-(Isobutoxymethyl)acrylamide
**iBuMA**	173.5	210.5	Isobutyl methacrylate
**iCEMA**	30.6	49.0	Isocyanatoethyl methacrylate

**iDA**	251.1	13.8	Isodecyl acrylate
**iDMA**	177.6	60.6	Isodecyl methacrylate
**iOA**	167.4	109.5	Isooctyl acrylate
**iPAM**	71.4	88.6	N-Isopropylacrylamide
**LaA**	28.6	131.0	Lauryl acrylate
**LMA**	153.1	69.0	Lauryl methacrylate
**MA**	157.2	102.4	Methyl acrylate
**MAA**	24.5	146.4	Methyl 2-acetamidoacrylate
**MAAH**	28.6	100.4	Methacrylic anhydride
**MAAHS**	281.7	164.3	Methacrylic acid N-hydroxysuccinimide ester
**MAEA**	42.9	54.4	Methacryloyloxy)ethyl acetoacetate
**MAEACl**	385.8	287.3	[2-(Methacryloyloxy)ethyl]trimethylammonium chloride solution
**MAEP**	32.7	288.7	Monoacryloxyethyl phosphate
**MAEPC**	14.3	198.6	2-Methacryloyloxyethyl phosphorylcholine
**MAETA**	161.3	127.2	4-Methacryloxyethyl trimellitic anhydride
**MAHBP**	40.8	37.1	4-Methacryloxy-2-hydroxybenzophenone
**MAL**	434.8	226.6	Methacryloyl-L-Lysine
**Mam**	157.2	124.9	Methacrylamide
**MAPtMA**	330.7	332.2	Methacrylamidopropyltrimethylammonium chloride,
**MAPU**	0.0	0.0	2-methacryloxyethyl phenyl urethane
**MBAm**	312.3	1.1	N,N'-Methylenebisacrylamide
**MBMAm**	573.6	373.1	N,N'-Methylenebismethacrylamide
**MEDMSAH**	8.2	132.9	[2-(Methacryloyloxy)ethyl]dimethyl-(3-sulfopropyl) ammonium hydroxide
**MHMB**	38.8	113.4	Methyl 3-hydroxy-2-methylenebutyrate
**MMA**	120.4	65.5	Methyl methacrylate
**MMAm**	34.7	176.5	N-Methylmethacrylamide
**mMAOEM**	253.1	103.2	mono-2-(Methacryloyloxy)ethyl maleate
**mMAOES**	36.7	99.4	mono-2-(Methacryloyloxy)ethyl succinate
**MOPAm**	132.7	39.4	N-(3-Methoxypropyl)acrylamide
**MPDSAH**	26.5	160.1	Methacryloylamino)propyl]dimethyl(3-sulfopropyl)ammonium hydroxide inner salt
**MTEMA**	216.4	49.1	Methylthioethyl methacrylate
**NAM**	196.0	230.9	N-Acryloylmorpholine
**NaPhA**	104.1	292.7	Sodium 3-phenyl-acrylate
**NAS**	404.2	256.4	N-Acryloxysuccinimide
**NBMA**	26.5	167.8	Norbornyl methacrylate
**NBnMA**	434.8	850.8	o-Nitrobenzyl methacrylate, . 95%
**NDDMA**	32.7	98.7	1,9-Nonanediol dimethacrylate
**NDMAm**	118.4	122.2	N-Dodecylmethacrylamide
**NGDA**	75.5	71.3	Neopentyl glycol diacrylate
**NGPDA**	87.8	105.8	Neopentyl glycol propoxylate diacrylate
**NMEMA**	440.9	301.1	2-N-Morpholinoethyl methacrylate, 95%
**nOcMA**	81.6	158.1	n-Octyl methacrylate,
**NpA**	44.9	41.8	Naphthyl acrylate
**NPhPMA**	473.6	901.2	Nitrophenyl-2-pyrrolidonemethyl acrylate
**NpMA**	4.1	1.1	Naphthyl methacrylate
**ODA**	95.9	112.6	Octadecyl acrylate
**OFHMA**	430.7	222.5	Octafluoro-2-hydroxy-6-(trifluoromethyl)heptyl methacrylate
**OFPA**	57.2	94.1	Octafluoropentyl acrylate
**OFPMA**	22.5	128.8	Octafluoropentyl methacrylate
**PA**	357.2	138.5	Propargyl acrylate
**PAHEMA**	200.0	240.4	Phosphoric acid 2-hydroxyethyl methacrylate ester
**PBBA**	140.8	14.3	Pentabromobenzyl acrylate
**PBPhA**	98.0	29.9	Pentabromophenyl acrylate

**PBPhMA**	161.3	41.0	Pentabromophenyl methacrylate
**PDA**	24.5	41.8	1,4-Phenylene diacrylate
**PDDMA**	46.9	135.7	1,5-Pentanediol dimethacrylate
**PEDAM**	26.5	143.1	Pentaerythritol diacrylate monostearate
**pEGDA**	106.1	61.4	Polyethylene glycol diacrylate
**pEGDMA**	65.3	82.6	Poly(ethylene glycol) (600) dimethacrylate
**pEGMA**	81.6	85.2	Poly(ethylene glycol) methacrylate
**pEGMEA**	230.7	40.1	Poly(ethylene glycol) methyl ether acrylate
**pEGMEMA**	30.6	67.2	Poly(ethylene glycol) methyl ether methacrylate
**pEGPhEA**	42.9	128.9	Poly(ethylene glycol) phenyl ether acrylate
**PETA**	63.3	53.5	Pentaerythritol tetraacrylate
**PETrA**	110.2	36.5	Pentaerythritol triacrylate
**pFDA**	185.8	171.9	Perfluorodecyl acrylate
**PFPA**	55.1	82.2	Pentafluoropropyl acrylate
**PFPhA**	261.3	462.5	Pentafluorophenyl acrylate
**PFPhMA**	134.7	84.0	Pentafluorophenyl methacrylate, 95%
**PFPMA**	20.4	73.6	Pentafluoropropyl methacrylate
**PhA**	183.7	54.5	Phenyl acrylate, 95%
**PhEA**	87.8	93.7	2-Phenylethyl acrylate
**PhEMA**	8.2	128.3	2-Phenylethyl methacrylate
**PhMA**	2.0	86.7	Phenyl methacrylate
**PhMAm**	69.4	14.9	N-Phenylmethacrylamide
**PHPMA**	24.5	19.8	3-Phenoxy 2 hydroxy propyl methacrylate
**PMA**	149.0	134.6	Propargyl methacrylate
**PMAm**	0.0	250.9	N-(Phthalimidomethyl)acrylamide
**PMMA**	342.9	466.7	1-Pyrenylmethyl methacrylate
**pPGA**	71.4	77.8	Poly(propylene glycol) acrylate
**pPGDA**	91.9	43.1	Poly(propylene glycol) diacrylate
**pPGDMA**	169.4	46.9	Poly(propylene glycol) (400) dimethacrylate
**pPGMEA**	38.8	86.0	Poly(propylene glycol) methyl ether acrylate
**pPGNEA**	49.0	81.8	Poly(propylene glycol) 4-nonylphenyl ether acrylate
**PPPDMA**	59.2	131.2	PEO(5800)-b-PPO(3000)-b-PEO(5800) dimethacrylate
**SEMA**	0.0	97.1	2-Sulfoethyl methacrylate
**SMA**	167.4	194.0	Stearyl methacrylate
**SPAK**	353.1	113.9	Sulfopropyl acrylate potassium salt
**SPMAK**	24.5	92.6	3-Sulfopropyl methacrylate potassium salt
**TAHTA**	220.5	211.5	1,3,5-Triacryloylhexahydro-1,3,5-triazine
**TAIC**	222.5	73.1	Tris[2-(acryloyloxy)ethyl] isocyanurate
**tBA**	144.9	98.1	Tert-butyl acrylate
**tBAEMA**	183.7	2051.4	Tert-butylamino-ethyl methacrylate
**tBAm**	28.6	111.1	N-tert-Butylacrylamide
**tBCHA**	59.2	53.2	Tert-butylcyclohexylacrylate
**tBCHMA**	89.8	43.8	Tertbutylcyclohexyl methacrylate
**tBEMAm**	106.1	69.8	N-(3,3-dimethylbutyl)methacrylamide
**tBMA**	226.6	132.4	Tert-butyl methacrylate
**tBMAm**	106.1	42.4	N-tert-Butylmethacrylamide
**TBNpMA**	73.5	53.1	Tribromoneopentyl methacrylate
**tBOCAPAm**	112.3	63.0	N-(t-BOC-aminopropyl)methacrylamide
**TBPhA**	144.9	83.0	2,4,6-Tribromophenyl acrylate
**TBPMA**	151.1	79.8	Tribromophenyl methacrylate
**TCDMDA**	46.9	32.2	Tricyclodecane-dimethanol diacrylate
**TDFOcA**	142.9	475.3	Tridecafluorooctyl acrylate
**TDFOMA**	149.0	134.2	Tridecafluorooctyl methacrylate
**TEGDA**	65.3	86.9	Tetra(ethylene glycol) diacrylate

**TEGMA**	40.8	28.5	Tri(ethylene glycol) methyl ether methacrylate
**TFCAm**	316.4	88.7	7-[4-(Trifluoromethyl)coumarin]acrylamide
**TFPMA**	138.8	563.5	Tetrafluoropropyl methacrylate
**THFuA**	73.5	122.9	Tetrahydrofurfuryl acrylate
**THFuMA**	36.7	97.8	Tetrahydrofurfuryl methacrylate
**THMMAm**	44.9	363.8	N-[Tris(hydroxymethyl)methyl]acrylamide
**TMBAm**	128.6	56.6	N-(1,1,3,3-Tetramethylbutyl)acrylamide
**TMCHMA**	193.9	69.5	Trimethylcyclohexyl methacrylate
**TMHA**	67.4	107.1	Trimethylhexyl acrylate
**TMOBDA**	69.4	40.2	Trimethylolpropane benzoate diacrylate
**TMOPTMA**	165.3	33.5	1,1,1-Trimethylolpropane trimethacrylate
**TMPDAE**	42.9	62.3	Trimethyl propane diallyl ether
**TMPETA**	49.0	26.4	Trimethylolpropane ethoxylate triacrylate
**TMPOTA**	163.3	38.5	Trimethylolpropane propoxylate triacrylate
**TMPTA**	61.2	9.0	Trimethylolpropane triacrylate
**TPGDA**	57.2	15.4	Tri(propylene glycol) diacrylate
**TPhMAm**	83.7	41.9	N-(Triphenylmethyl)methacrylamide
**VMA**	183.7	126.3	Vinyl methacrylate
**ZnA**	8.2	177.8	Zinc acrylate
**ZrA**	191.9	439.8	Zirconium acrylate
**ZrBNCTA**	26.5	185.8	Zirconium bromonorbornanelactone carboxylate triacrylate
**ZrCEA**	30.6	546.0	Zirconium carboxyethyl acrylate

[a]Arbitrary units of fluorescence; higher value indicates greater attachment.

Table S2. Identities and structures of polymers resistant to colonization by *C. albicans*

Acronym	Full name	Biofilm formation after 24 h (%)[a]	WCA (°)[b]
**AODMBA**	(R)-α-Acryloyloxy-β,β-dimethyl-γ-butyrolactone	4.1 ± 3.0	67.1 ± 19.9
**CNEA**	Cyanoethyl acrylate	3.1 ± 1.6	71.8 ± 10.1
**tBCHMA**	Tertbutylcyclohexyl methacrylate	13.6 ± 9.6	89.6 ± 6.1
**PDA**	1,4-Phenylene diacrylate	1.0 ± 0.2	62.4 ± 3.0
**DEGMA**	Di(ethylene glycol) methyl ether methacrylate	16.3 ± 11.3	47.9 ± 15.6
**TEGMA**	Tri(ethylene glycol) methyl ether methacrylate	13.0 ± 6.7	23.7 ± 3.52
**PHPMA**	3-Phenoxy 2 hydroxy propyl methacrylate	18.4 ± 3.2	69.4 ± 2.76
**tBCHA**	Tert-butylcyclohexylacrylate	18.5 ± 4.5	96.1 ± 6.07
**iDMA**	Isodecyl methacrylate	19.1 ± 2.0	75.4 ± 5.3

[a]Mean value from at least three independent experiments ±SEM; according to XTT signal as a percentage of the signal obtained in non-coated wells. Polymers shown are those giving <25% attachment.
[b]Water contact angle; mean value from three independent experiments ±SD.

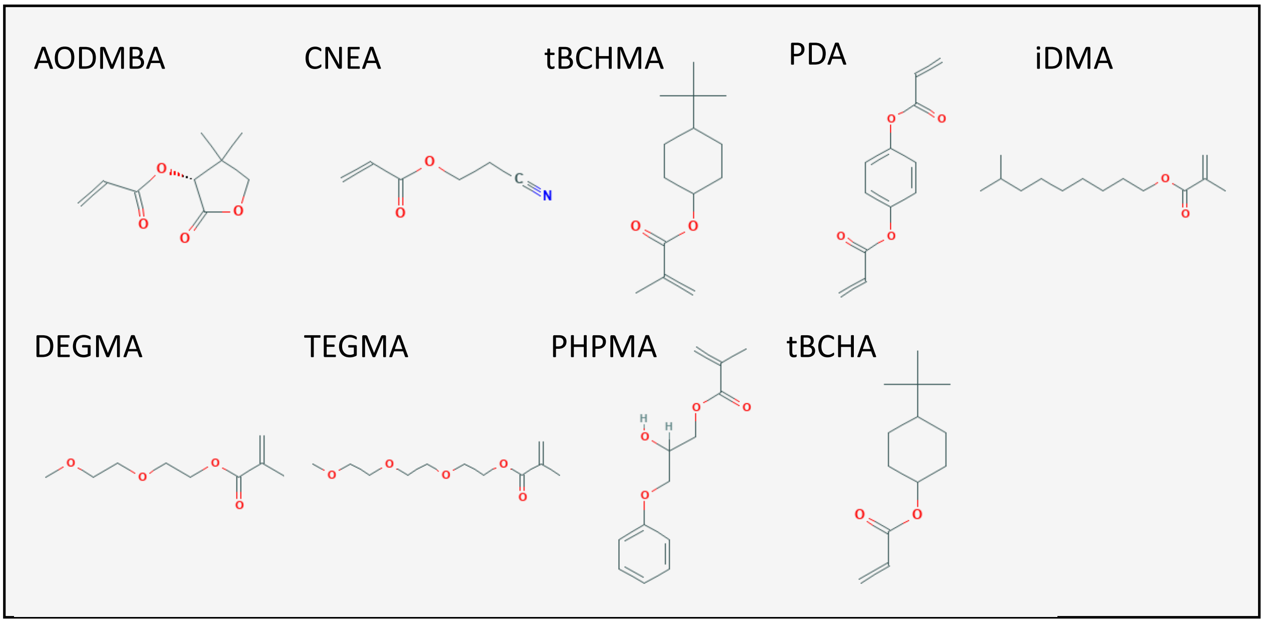

Table S3. Identities and structures of polymers resistant to colonization by filamentous fungi

Acronym	Full name	Biofilm formation - 24 h (%)[a]			WCA (°)[b]
		B.cinerea	*S.tritici*	*A.brasiliensis*	
**ZrCEA**	Zirconium carboxyethyl acrylate	1.0 ± 0.3	0.6 ± 0.1	2.7 ±1.4	20.8 ± 3.6
**HfCEA**	Hafnium carboxyethyl acrylate	1.1 ± 0.8	0.9 ± 0.5	1.4 ± 0.7	23.4 ± 6.5
**PDA**	1,4-Phenylene diacrylate	1.2 ± 0.3	0.2 ± 0.1	0.6 ± 0.1	62.4 ± 2.9
**DEAEA**	Diethylamino ethyl acrylate	1.3 ± 1.1	3.9 ± 3.8	0.2 ± 0.2	47.8 ± 13.0
**mMAOES**	mono-2-(Methacryloyloxy)ethyl succinate	1.4 ± 0.6	0.9 ± 0.8	0.2 ± 0.2	50.5 ± 4.8
**MAA**	Methyl 2-acetamidoacrylate	2.8 ± 1.1	6.2 ± 2.8	6.3 ± 4.7	52.9 ± 15.7
**TEGMA**	Tri(ethylene glycol) methyl ether methacrylate	6.6 ± 1.9	4.3 ± 4.1	6.4 ± 2.4	23.7 ± 3.5
**DiPEMA**	2-Diisopropylaminoethyl methacrylate	7.4 ± 0.4	23.3 ± 6.1	25.4 ± 1.3	53.4 ± 6.7
**pEGPhEA**	Poly(ethylene glycol) phenyl ether acrylate	9.1 ± 1.0	11.5 ± 1.5	15.6 ± 2.4	33.2 ± 5.8
**MAAH**	Methacrylic anhydride	9.2 ± 7.4	6.1 ± 4.3	1.8 ± 1.8	49.2 ± 9.2
**DEGEEA**	Di(ethylene glycol) ethyl ether acrylate	10.3 ± 3.8	0.9 ± 0.8	7.7 ± 5.8	46.4 ± 12.2
**EOEA**	Ethoxyethyl acrylate	13.7 ± 1.3	8.1 ± 3.8	14.6 ± 8.2	41.7 ± 1.7
**PMAm**	N-(Phthalimidomethyl)acrylamide	14.0 ± 4.2	46.6 ± 7.3	4.6 ± 0.5	67.1 ± 13.3
**tBAm**	N-tert-Butylacrylamide	15.0 ± 5.7	28.5 ± 10.7	39.5 ± 6.9	53.0 ± 15.6
**LaA**	Lauryl acrylate	15.1 ± 10	16.5 ± 3.0	22.5 ± 9.1	43.4 ± 8.3
**DEGMA**	Di(ethylene glycol) methyl ether methacrylate	16.7 ± 4.2	3.9 ± 0.1	19.6 ± 5.0	47.9 ± 15.6
**CNEA**	Cyanoethyl acrylate	18.2 ± 5.3	36.5 ± 1.9	27.3 ± 5.2	71.8 ± 10.1
**CMAOE**	Caprolactone 2-(methacryloyloxy)ethyl ester	19.2 ± 8.4	33.7 ±5.0	33.2 ±5.7	34.8 ± 4.0
**EGMEA**	Ethylene glycol methyl ether acrylate	23.0 ± 1.2	5.7 ±0.5	20.4 ±4.5	55.2 ± 11.8

[a]Mean value from at least three independent experiments ±SEM; according to XTT signal as a percentage of the signal obtained in non-coated wells. Polymers shown are those giving <25% attachment with *B. cinerea.*
[b]Water contact angle; mean value from three independent experiments ±SD.

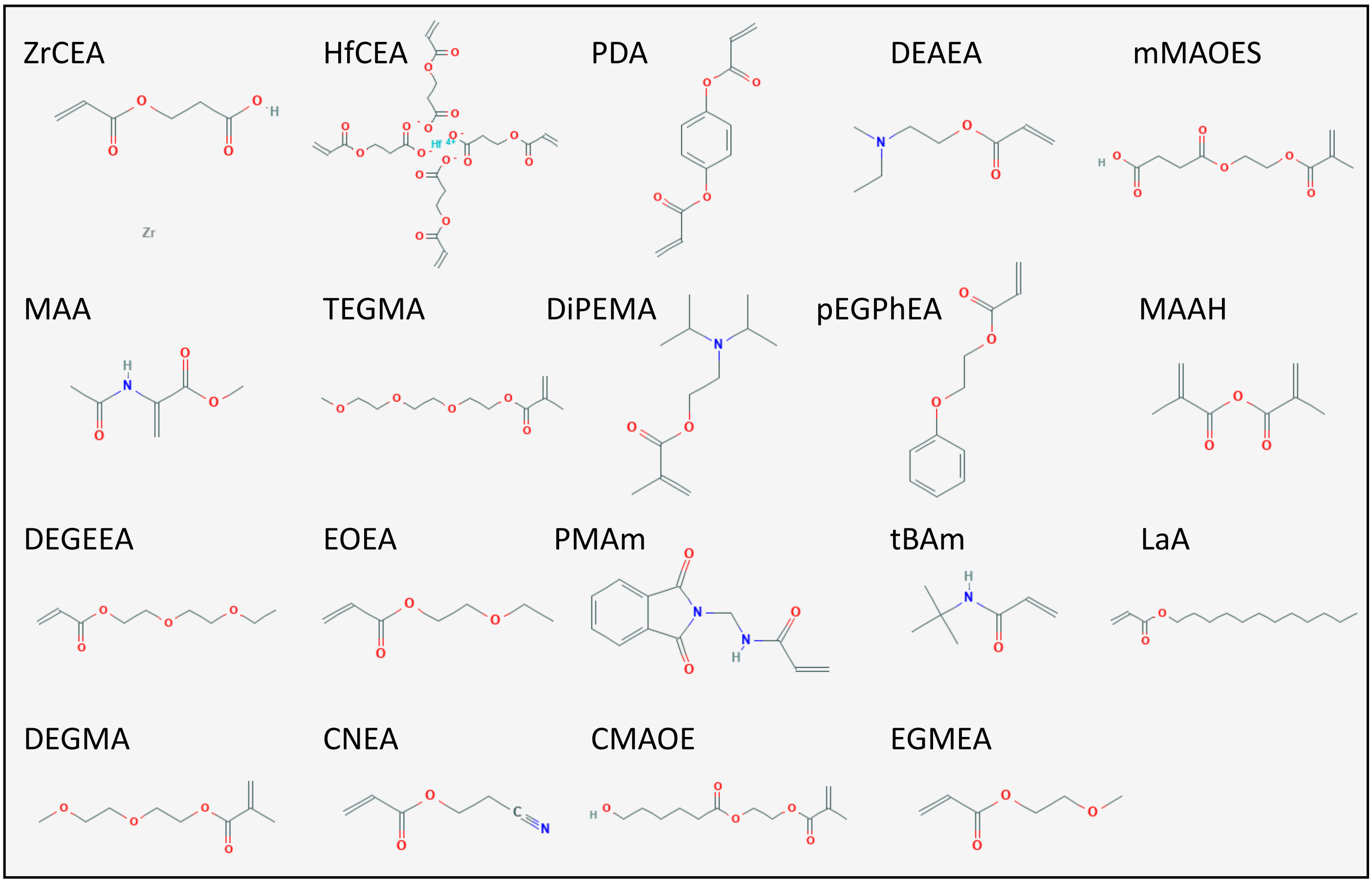

Table S4. Synthesis of the fungal anti-attachment materials by free radical polymerisation using a thiol chain transfer agent, for coating leaves. Percentage of conversion of (meth)acrylate monomers to cured polymers, molecular weight (number average, M_n) and polydispersity (Đ) for each material were determined by GPC and ^{1}H-NMR analysis. ^{1}H-NMR spectra are presented beneath the table.

Acronym	% conversion	M_n (Da, GPC)	Đ	M_n (Da, ^{1}H-NMR)
DEGEEA	>90	4202	1.27	5096
DEGMA	>90	5778	1.40	6154
EGMMA	90	4197	1.46	2951
TEGMA	>90	7246	1.41	4231
mMAOES	80-85%	-	-	8464

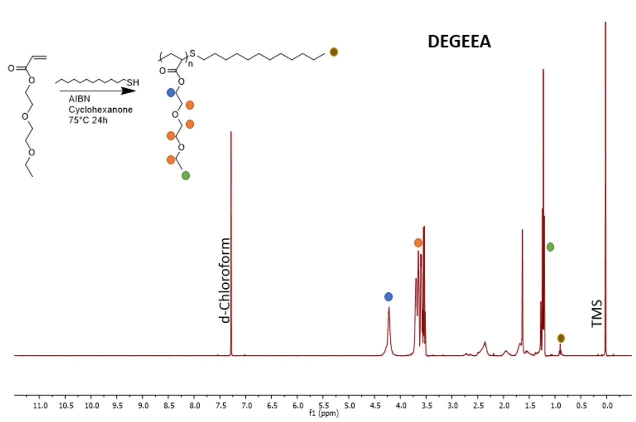

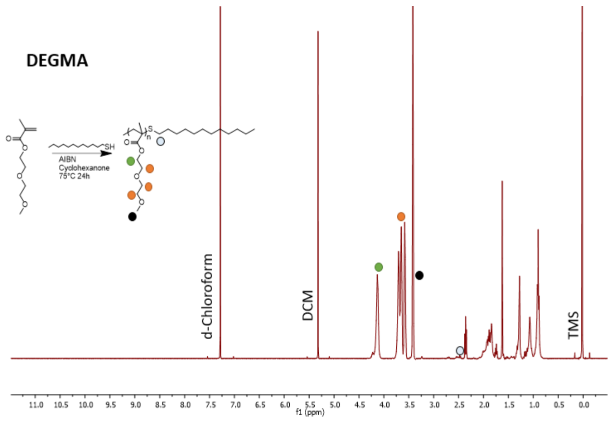

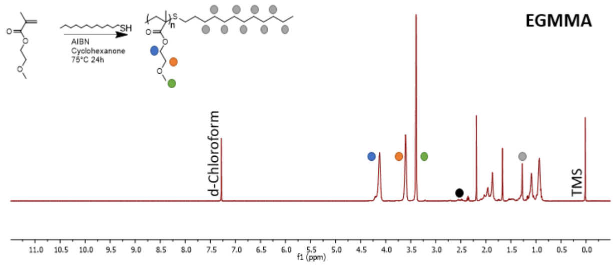

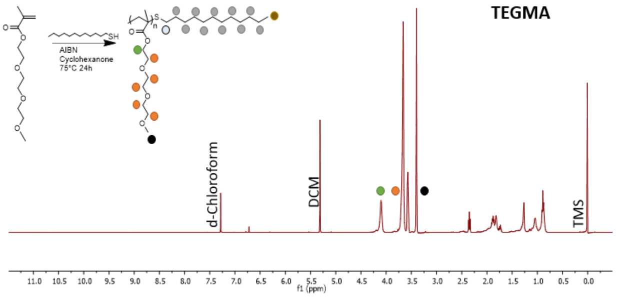

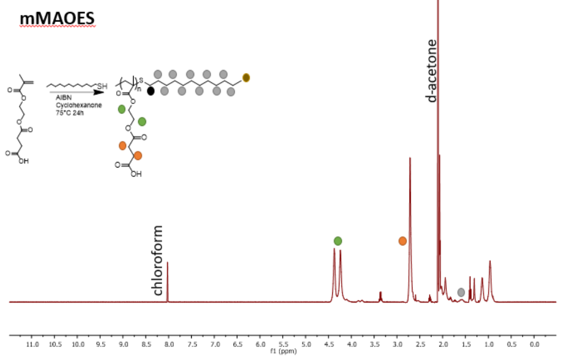

A

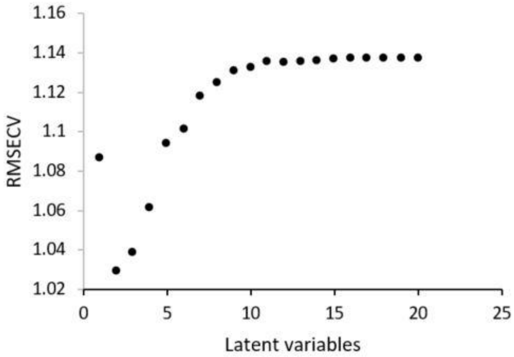

C

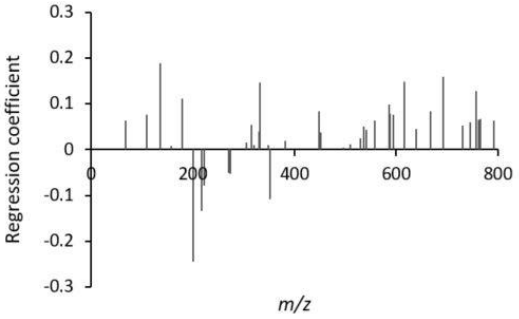

B

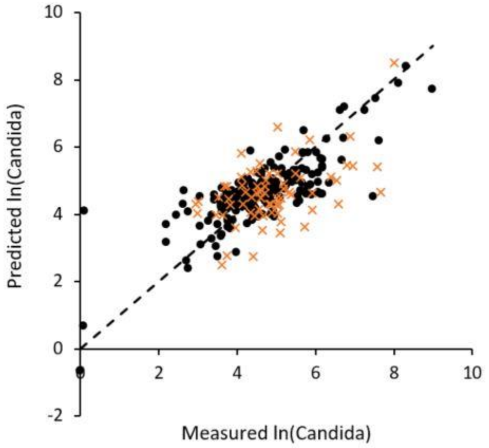

D

RC	m/z	Possible Assignment 1	2	3	4	5
0.19	95.9455	SO_4^-				
0.16	84.0698	$C_4H_8N_2^+$				
0.15	42.0372	$C_2H_4N^+$				
0.15	272.7892	$C_2SO_{10}F_3^-$	$C_4SNO_{10}F^-$			
0.13	136.0279	$C_6H_4N_2O_2^+$	$C_3H_6NO_5^+$	$C_{11}H_4^+$	$C_4H_8O_5^+$	$C_7H_4O_3^+$
0.10	336.1276	$C_{23}H_{16}N_2O^+$	$C_{21}H_{20}O_4^+$	$C_{24}H_{18}NO^+$	$C_{24}H_{16}O_2^+$	$C_{25}H_{20}O^+$
-0.24	144.0016	C_{12}^-	$C_9H_4S^-$	$C_4H_4SN_2O_2^-$	$C_5H_4O_5^-$	$C_8H_2NO_2^-$
-0.13	160.0109	$C_8H_4SN_2^-$	$C_{11}N_2^-$	$C_9H_4O_3^-$	$C_5H_4O_6^-$	
-0.13	255.0509	$C_{18}H_7O_2^+$	$C_{15}H_{11}O_4^+$	$C_{18}H_9NO^+$	$C_{14}H_7O_5^+$	
-0.12	787.1606	$C_{41}H_{41}O_4F_{10}^+$	$C_{42}H_{42}O_2F_{11}^+$	$C_{43}H_{43}F_{12}^+$		
-0.11	291.0426	$C_{17}H_7O_5^-$	$C_{18}H_{11}O_4^-$	$C_{21}H_9NO^-$	$C_{16}H_5NO_5^-$	

Fig. S1. PLS regression correlating the natural log of fluorescence due to *C. albicans* attachment and surface chemistry as measured by ToF-SIMS. (**A**) RMSECV for PLS regression conducted with varying numbers of latent variables. Three latent variables were selected for the final PLS model. (**B**) The measured versus predicted fungal attachment values for the training (l) and test (**×**) datasets. The y = x line is drawn as a guide. R^2 = 0.43. (**C**) The regression coefficients for the X-variables after sparse selection. A total of 37 variables were used for the model. (**D**) List of the top ions with the largest positive or negative regression coefficients used in the PLS model with possible chemical assignments.

A weak correlation was observed by PLS models between surface chemistry as represented by ToF-SIMS spectra and the attachment of *C. albicans* or *B. cinerea* (R^2 = 0.43 and 0.20, respectively) (Figs. S1-2). Thus, although surface chemistry contributes to the attachment of the fungi to the different polymers, other variables such as cell or spore morphology and the polymers' physical form also likely play a significant role in determining the extent of attachment. Ions likely associated with nitrate and sulphate groups were assigned positive regression coefficients for models generated for both fungal species, suggesting these molecular groups are associated with high fungal attachment, possibly through an electrostatic interaction. Conversely, aliphatic carbon and high oxygen containing fragments were assigned low regression coefficients in the PLS models for both fungal species. Using non-linear ML improved the predictive power of the models compared with PLS regression for both fungal species (Fig. S3). For *C. albicans* the R^2 value increased from 0.43 to 0.47 and for *B. cinerea* the R^2 value increased from 0.20 to 0.35. The small increase suggests that the relationship between fungal attachment and surface chemistry was largely linear in nature.

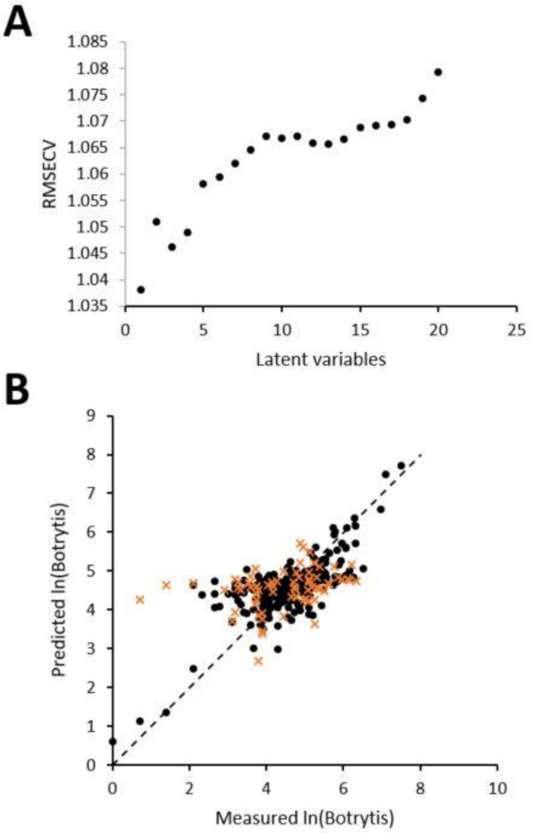

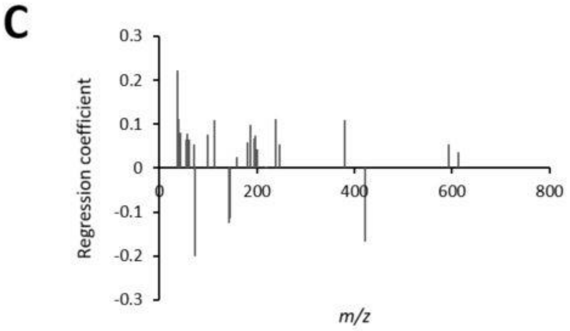

D

RC	m/z	Possible Assignment 1	2	3	4	5
0.22	37.1571	C_3H^-				
0.11	39.9878	C_2O^+				
0.11	238.1116	$C_{15}H_{14}N_2O^+$	$C_{12}H_{16}NO_4^+$	$C_{13}H_{18}O_4^+$	$C_{16}H_{16}NO^+$	$C_{16}H_{14}O_2^+$
0.11	380.0817	$C_{25}H_{16}SO_2^-$	$C_{21}H_{16}O_7^-$	$C_{25}H_{16}O_4^-$	$C_{21}H_{18}NO_6^-$	
0.11	112.0366	$C_6H_8S^+$	$C_5H_6NO_2^+$	$C_9H_4^+$	$C_4H_4N_2O_2^+$	
0.10	186.0049	$C_9H_2N_2O_3^-$	$C_7H_6SO_4^-$	$C_{11}H_6SO^-$	$C_{10}H_2O_4^-$	$C_{10}H_4NO_3^-$
0.08	42.0372	$C_2H_4N^+$				
0.08	56.0174	$C_2H_2NO^+$				
0.08	97.976	C_3SNO^-	$C_4H_2SO^-$			
-0.20	72.0305	$C_2H_4N_2O^+$				
-0.17	421.0839	$C_{22}H_{15}NO_8^-$	$C_{23}H_{17}O_8^-$	$C_{22}H_{13}O_9^-$	$C_{23}H_{19}NO_7^-$	
-0.12	143.0054	$C_4H_3N_2O_4^-$	$C_8HNO_2^-$	$C_5H_3O_5^-$	$C_9H_3O_2^-$	$C_9H_3S^-$
-0.12	144.0016	C_{12}^-	$C_9H_4S^-$	$C_5H_4O_5^-$	$C_8H_2NO_2^-$	$C_4H_2NO_5^-$

Fig. S2. PLS regression correlating the natural log of fluorescence due to *B. cinerea* attachment and surface chemistry as measured by ToF-SIMS. (**A**) RMSECV for PLS regression conducted with varying numbers of latent variables. Three latent variables were selected for the final PLS model. (**B**) The measured versus predicted fungal attachment values for the training (l) and test (**×**) datasets. The y = x line is drawn as a guide. R^2 = 0.20. (**C**) The regression coefficients for the X-variables after sparse selection. A total of 24 variables were used for the model. (**D**) List of the top ions with the largest positive or negative regression coefficients used in the PLS model with possible chemical assignments.

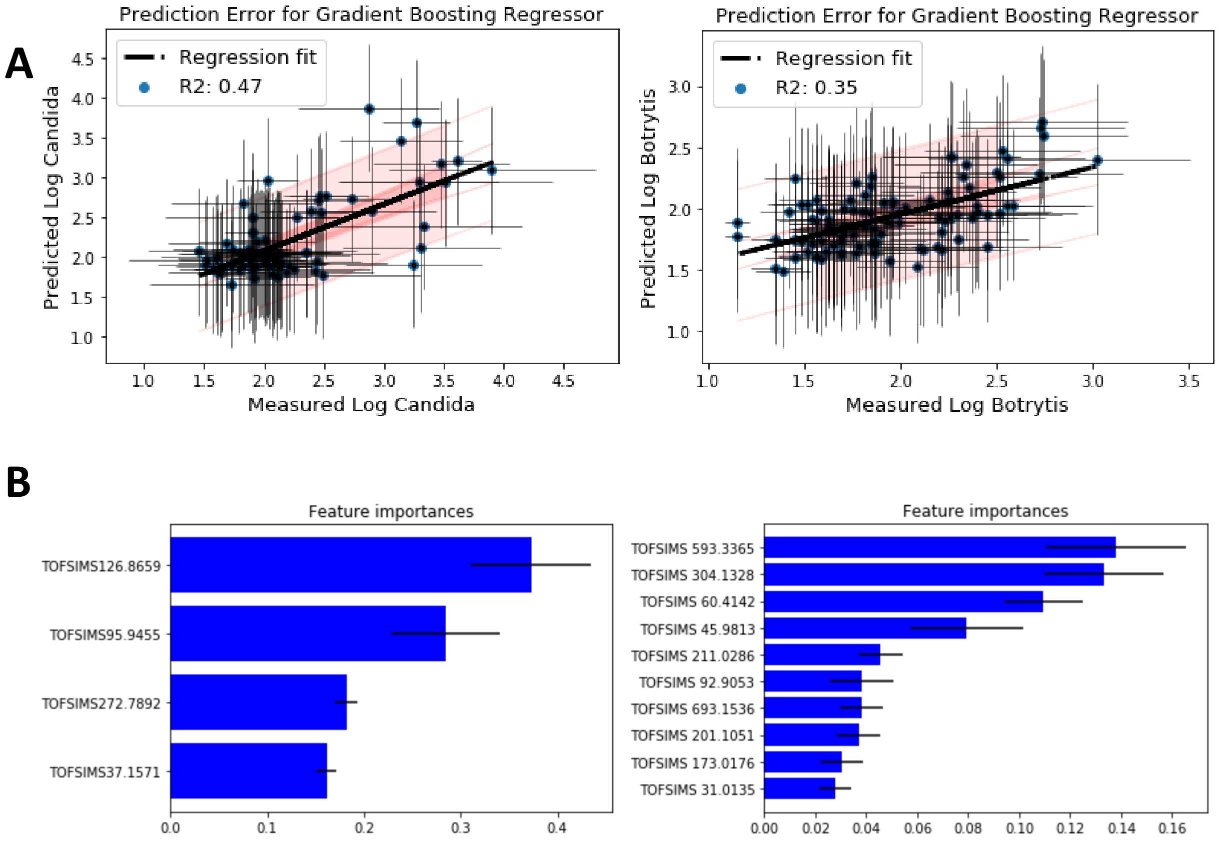

Fig. S3. Machine learning (ML) model correlating the natural log of fluorescence due to *C. albicans* (left panels) or *B. cinerea* (right) attachment and surface chemistry as measured by ToF-SIMS. (**A**) The measured versus predicted fungal attachment values with standard deviation of measured and predicted values and prediction confidence levels. The y = x line is drawn as a guide. R^2 = 0.47 (*C. albicans*) and 0.35 (*B. cinerea*). (**B**) Ions associated with high or low feature importance. Left panel, *C. albicans*; Right panel, *B. cinerea.*

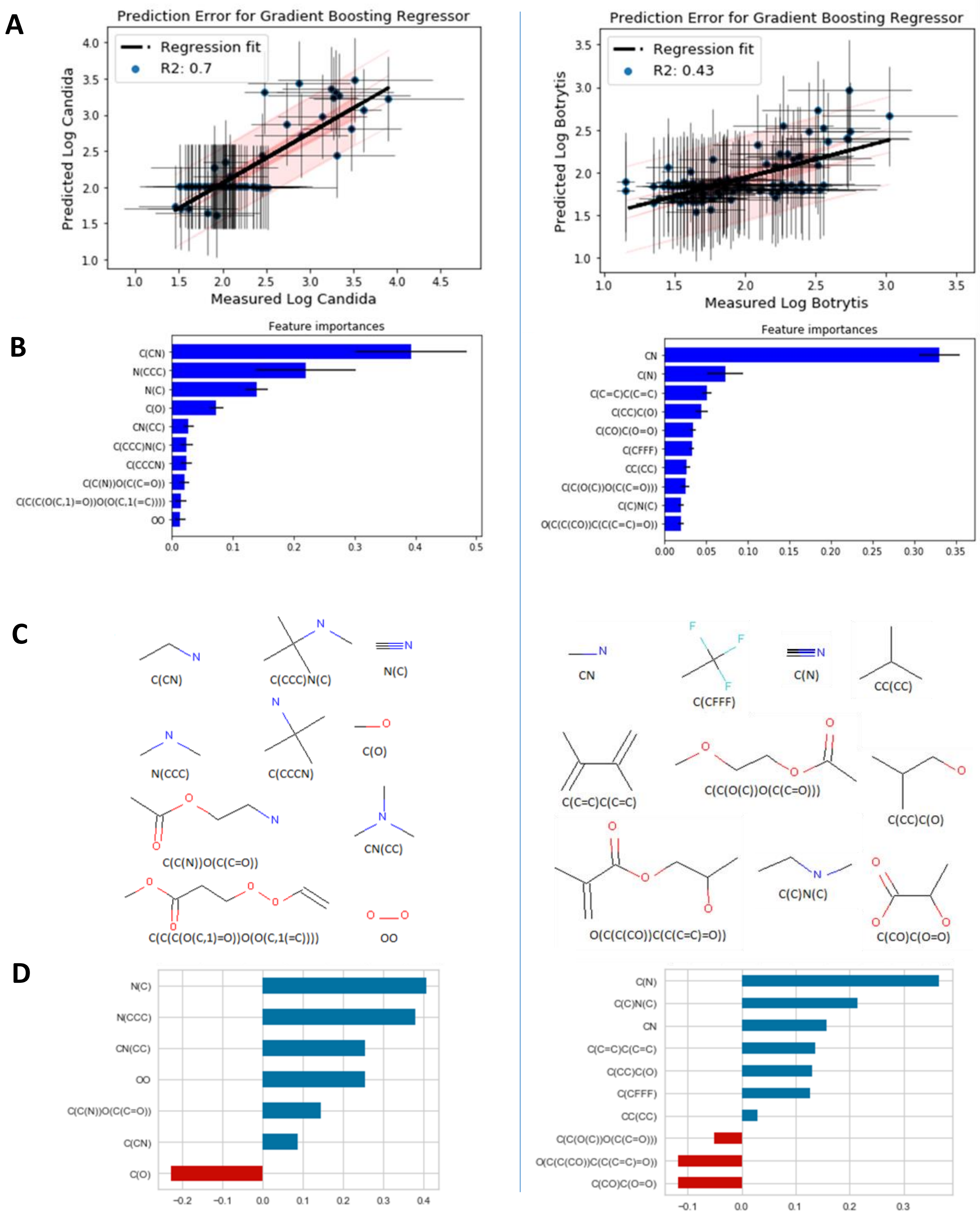

Fig. S4. ML results for *C. albicans* (left panels) or *B. cinerea* (right*)* using signature molecular descriptors. (**A**) Measured versus predicted attachment XGBoost regression results with standard deviation of measured and predicted values and prediction confidence levels. The y = x line is drawn as a guide. R^2 = 0.70 *(C. albicans*, left) or 0.43 (*B. cinerea*, right). (**B**) Ranked feature importance, with the most relevant fragment descriptors selected for modelling. (**C**) Molecular fragments most relevant to attachment. (**D**)

Coefficients for key ions taken from a multiple linear regression model with expectation maximisation indicating whether an ion was positively or negatively correlated with fungal biofilm formation. Coefficients have not been shown for some descriptors identified as important in the model generated from the machine learning approach where the polarity was not clear. This is likely due to the non-linear nature of the machine learning model and the multiple linear regression approach required for determining these coefficients, suggesting that the correlation of these descriptors with fungal biofilm is non-linear in nature.

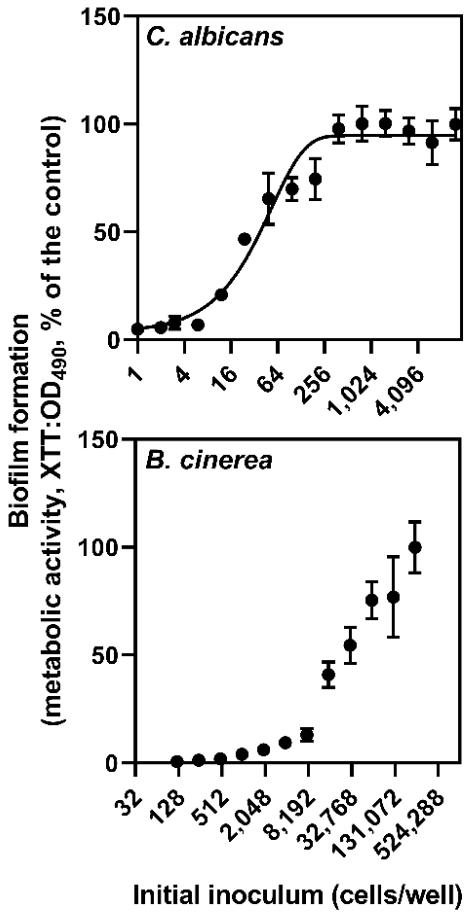

Fig. S5. Relationship between inoculum-size and subsequent biofilm detection with the XTT assay (metabolic activity measurement). As depicted in Figure 2A, non-coated wells were inoculated with different concentrations of *C. albicans* cells or *B. cinerea* spores for 2 or 6 h, respectively. Non-adherent cells were washed away and fresh medium was added to the wells. After 24 h, wells were washed again and biofilm formation assessed using XTT salt. The values are means ±SEM from at least three replicate experiments. In the screens performed during this work, materials-of-interest were designated as those yielding a biofilm (metabolic activity) < 25% compared to the control. In the case of *C. albicans*, Figure S5 shows that < 25% is approximately equivalent to a biofilm that would be formed from a starting inoculum of ≤ 10 cells. That is, since the starting inoculum used in the main assays is ~12,500 cells of *C. albicans* per well, ~10 cells equates to a ~99.9% reduction in attachment (bearing in mind that biofilm formation is saturated at a starting inoculum above ~250 cells; top panel). In the case of *B. cinerea*, < 25% is approximately equivalent to a biofilm that would be formed from a starting inoculum of ≤ 10,000 spores; as the starting inoculum used in the main assays is ~250,000 *B. cinerea* spores/well, ~10,000 spores equates to a ~96% reduction in attachment.

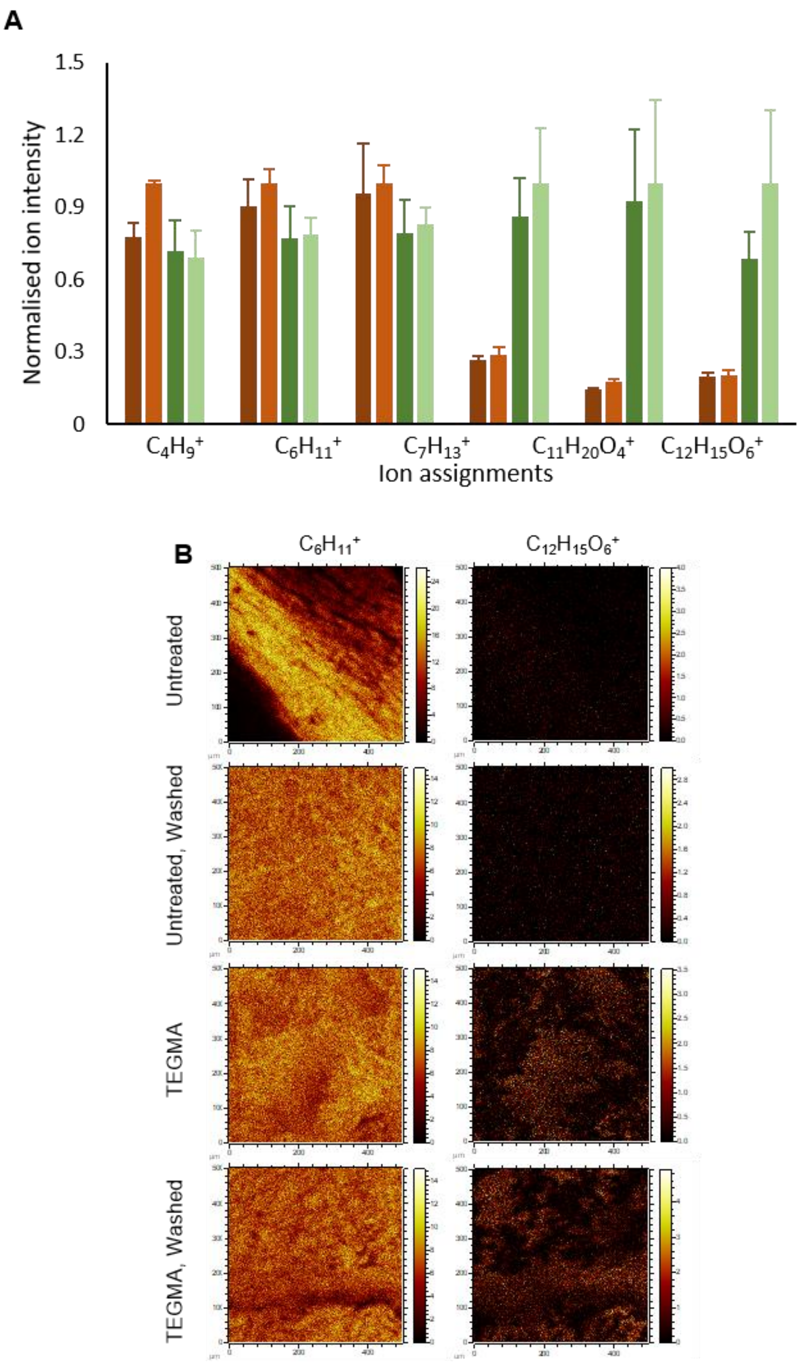

Fig. S6. Retention of TEGMA on leaves after washing. (**A**) Leaf sections untreated (n) or coated with TEGMA (n) and either unwashed (dark) or washed (light) were assessed by ToF-SIMS using the same experimental conditions used to characterize polymer samples. Aliphatic carbon ions likely associated with wax on the leaf ($C_4H_9^+$, $C_6H_{11}^+$ and $C_7H_{13}^+$) or ions likely associated with the oligoethylene glycol moiety ($C_{11}H_{20}O_4^+$, $C_{12}H_{15}O_6^+$ and $C_{13}H_{29}O_7^+$) were quantified for each sample. Ion intensity was normalized to total ion count and then to the maximum intensity observed for each ion across the four sample sets. Error bars equal one standard deviation unit (N=3). Peaks associated with the oligoethylene moiety were significantly higher ($p<0.025$) on treated samples compared with untreated samples. No significant change was observed after washing. (**B**) Ion images for characteristic ions $C_6H_{11}^+$ (leaf) and $C_{12}H_{15}O_6^+$ (oligoethylene). The coating appears patchy on the TEGMA-treated sample, with regions associated with the polymer and other regions associated with the leaf both visible.
